# Seizure Occurrence After Solid Organ Transplantation: A Systematic Review and Meta-Analysis

**DOI:** 10.3390/jcm15218354

**Published:** 2026-10-27

**Authors:** Su-Jin Kang, Roo Ri Lee, Hyunkyung Jeon, Kiyon Rhew

**Affiliations:** 1 College of Pharmacy, Dongduk Women’s University, Seoul 02748, Republic of Korea; sjkang@dongduk.ac.kr (S.-J.K.); 20191804@dongduk.ac.kr (R.R.L.); 20191808@dongduk.ac.kr (H.J.); 2 Pharmaceutical Research Institute, Dongduk Women’s University, Seoul 02748, Republic of Korea

**Keywords:** solid organ transplantation, seizure, neurological complications, calcineurin inhibitor neurotoxicity, posterior reversible encephalopathy syndrome, systematic review, meta-analysis

## Abstract

**Background/Objectives:** Seizures are clinically important neurological complications after solid organ transplantation (SOT), but their reported frequency varies widely. We aimed to estimate the pooled seizure occurrence proportion after SOT and summarize reported seizure-related clinical factors. **Methods:** PubMed, Embase, and Web of Science were searched on 28 February 2026 for prospective or retrospective observational studies published in 2000–2025 that included SOT recipients and reported post-transplant seizures. No language restrictions were applied. Risk of bias was assessed using the Joanna Briggs Institute critical appraisal checklist for prevalence studies. Study-specific proportions were logit-transformed and pooled using random-effects models. Organ- and age-specific subgroup analyses, prediction intervals, sensitivity analyses, and assessments of small-study effects were performed. **Results:** Thirty-four reports representing 32 independent cohorts and 8816 recipients were included. The pooled seizure occurrence proportion was 5.3% (95% CI, 3.9–7.1%), with substantial heterogeneity (*I*^2^ = 87.0%) and a 95% prediction interval of 1.1–22.8%. Pooled seizure occurrence was higher in pediatric than adult cohorts (10.7% vs. 4.4%; between-group *p* = 0.011), although this study-level difference should not be interpreted as evidence of an independent effect of pediatric age. Commonly reported clinical contexts included calcineurin inhibitor-related neurotoxicity, metabolic or electrolyte disturbances, posterior reversible encephalopathy syndrome, cerebrovascular or structural brain injury, infection or systemic complications, and pre-existing neurological conditions. The evidence was limited by predominantly retrospective designs and heterogeneous seizure ascertainment and follow-up. **Conclusions:** The pooled study-specific seizure occurrence proportion after SOT was 5.3%, but occurrence varied substantially across populations and ascertainment periods, as reflected by the wide 95% prediction interval (1.1–22.8%). This estimate should not be interpreted as a uniform individual risk across transplant populations. Post-transplant seizures should prompt evaluation for potentially reversible and treatment-relevant causes. This review received no external funding and was not registered.

## 1. Introduction

Solid organ transplantation (SOT) has markedly improved patient survival and quality of life over the past two decades [1]. Advances in surgical techniques, perioperative care, and immunosuppressive therapies have substantially improved graft and patient survival across major transplant types [2,3]. As survival after transplantation continues to improve, the prevention and management of post-transplant complications have become increasingly important [4,5].

Despite these advances, post-transplant complications remain a major cause of morbidity and mortality. Neurological complications are particularly important because they may impair quality of life and prolong hospital and intensive care unit stays [6,7,8]. Seizures are among the most clinically important neurological complications after SOT, although their reported frequency varies substantially according to organ type, age group, follow-up duration, and ascertainment methods [9,10,11]. Clinical outcomes following seizures vary according to the underlying cause. Outcomes are generally favorable when reversible causes, such as electrolyte disturbances or drug toxicity, are identified; however, seizures associated with cerebrovascular disease, sepsis, or graft rejection often portend a poorer prognosis, particularly in liver transplant recipients [12,13].

Post-transplant seizures have a multifactorial etiology that includes calcineurin inhibitor (CNI) neurotoxicity, metabolic and electrolyte disturbances, infection, cerebrovascular or ischemic injury, and structural brain abnormalities [14,15,16,17,18,19,20,21]. Importantly, the occurrence of a post-transplant seizure does not by itself indicate epilepsy. Seizures occurring in the setting of acute metabolic, toxic, infectious, cerebrovascular, or other neurological insults may represent acute symptomatic events, whereas the development of recurrent unprovoked seizures or epilepsy represents a clinically distinct outcome. The relative contribution of these factors may differ according to the transplanted organ and the recipient’s clinical condition. In liver transplant recipients, seizures may occur in association with hepatic encephalopathy, graft dysfunction, rapid perioperative metabolic changes, infection, and immunosuppressant-related neurotoxicity. In kidney transplant recipients, electrolyte and osmotic shifts, hypertension, infection, and CNI neurotoxicity are frequently implicated. In heart transplant recipients, cerebrovascular events and ischemic injury appear to be particularly important, especially in patients with diabetes or renal dysfunction [12,15,16,17]. Tacrolimus and cyclosporine have been consistently associated with post-transplant neurotoxicity, including seizures and posterior reversible encephalopathy syndrome (PRES), through both dose-related and idiosyncratic mechanisms [18,19].

Previous studies have reported considerable variation in both the frequency and presumed causes of seizures after SOT, even within the same organ type. Some studies identified metabolic or electrolyte disturbances as the predominant contributors, whereas others emphasized immunosuppressant-related neurotoxicity [20,21,22]. Seizure frequency may also differ according to recipient age, with some studies suggesting a higher occurrence in pediatric than in adult recipients; however, estimates have been derived from heterogeneous populations and remain uncertain [23]. Although several reviews have addressed neurological complications after transplantation, no comprehensive meta-analysis has specifically estimated the pooled seizure occurrence proportion across solid organ transplant populations.

Therefore, we conducted a systematic review and meta-analysis to estimate the pooled seizure occurrence proportion after SOT and to descriptively summarize reported seizure-related clinical factors across solid organ transplant populations. This study aimed to provide quantitative and clinically relevant evidence to support risk stratification and the management of transplant recipients.

## 2. Materials and Methods

### 2.1. Literature Search

This systematic review and meta-analysis was reported in accordance with the Preferred Reporting Items for Systematic Reviews and Meta-Analyses 2020 statement (PRISMA 2020) [24]. The review was not registered, and no separate review protocol was prepared. PubMed, Embase, and Web of Science were searched on 28 February 2026, for studies published between 1 January 2000 and 31 December 2025. No separate gray-literature search or backward or forward citation searching was performed. No language restrictions were applied. The search strategy combined controlled vocabulary terms, where applicable, with text words related to seizures, neurological complications, and solid organ transplantation. The complete search strategies for each database are provided in Supplementary Table S2.

### 2.2. Eligibility Criteria and Study Selection

Studies were eligible for the systematic review if they: (1) included recipients of solid organ transplantation; (2) used a prospective or retrospective observational design; and (3) reported post-transplant seizure occurrence or seizure-related clinical factors.

For inclusion in the quantitative synthesis, studies were required to provide sufficient data to determine both the number of transplant recipients who experienced at least one seizure after transplantation and the corresponding number of transplant recipients at risk. Studies primarily focused on PRES or seizures were included in the quantitative synthesis only if they reported seizure occurrence in a defined underlying transplant cohort rather than exclusively among selected symptomatic patients. Studies that reported seizure-related clinical factors without an extractable numerator and denominator were included only in the qualitative synthesis.

Reports were excluded if they did not provide seizure-specific outcomes, focused exclusively on treatment outcomes, or reported only composite postoperative or neurological complications without separate seizure data. Case reports, case series without a defined underlying transplant cohort, reviews, editorials, conference abstracts, and previous meta-analyses were also excluded. The absence of an extractable transplant cohort denominator precluded inclusion in the quantitative synthesis but did not preclude qualitative inclusion when relevant seizure-related clinical factors were reported.

When two or more reports included the same or substantially overlapping study population, only one report was included in each quantitative analysis. The report with the largest cohort or the most complete seizure-specific outcome data was preferentially selected. Additional reports from overlapping populations were retained only when they provided relevant nonduplicative clinical information unavailable in the report selected for quantitative synthesis.

Two reviewers independently screened the titles and abstracts of retrieved records and subsequently assessed the full texts of potentially eligible reports. Disagreements regarding study eligibility were resolved through discussion and, when necessary, consultation with a third reviewer. No automation tools were used in the study-selection process.

### 2.3. Risk-of-Bias Assessment

The risk of bias of the included studies was assessed using the Joanna Briggs Institute critical appraisal checklist for studies reporting prevalence data because the primary outcome was the pooled proportion of post-transplant seizures [25]. The checklist consists of nine items addressing the sampling frame, participant selection, sample size, description of the study population and setting, validity and reliability of outcome measurement, statistical analysis, and adequacy of response or follow-up. Each item was rated as “yes,” “no,” or “unclear.”

Two reviewers independently assessed all nine items for each included report. Disagreements were resolved through discussion and, when necessary, consultation with a third reviewer. Item-level judgments constituted the primary risk-of-bias assessment. For descriptive reporting and sensitivity analyses, the review authors additionally applied an exploratory, review-specific operational classification according to the number of items rated ‘yes’: 7–9 items indicated low risk, 5–6 moderate risk, and four or fewer high risk. These cut-offs were used pragmatically to distinguish studies with most items rated ‘yes,’ an intermediate number of affirmative items, and fewer than half of the nine items rated ‘yes,’ respectively. They do not represent a validated overall risk-of-bias classification recommended by JBI. Studies were not excluded from the primary analysis solely on the basis of risk-of-bias classification.

### 2.4. Data Extraction

Two reviewers independently extracted data from the included studies using a standardized data extraction form. Discrepancies were resolved through discussion and, when necessary, consultation with a third reviewer.

Extracted study characteristics included the first author, publication year, country, study design, study period, study setting, and follow-up duration. Participant- and transplant-related characteristics included the transplanted organ, recipient age group, sample size, sex distribution, and donor type, when reported. Cohorts were classified as adult, pediatric, or mixed or insufficiently specified according to the age-group designation reported in the original study. We also recorded whether the study population was selected on the basis of seizures, PRES, or another neurological condition.

Seizure-related data included the number of recipients who experienced at least one post-transplant seizure, the total number of transplant recipients at risk, the seizure occurrence proportion, the definition and ascertainment method used for seizures, and the timing of seizure onset, when available. The outcome was assessed at the patient level, and recipients who experienced multiple seizure episodes were counted only once. Because definitions of acute symptomatic, unprovoked, early-onset, and late-onset seizures, status epilepticus, and subsequent epilepsy were not standardized across the included studies and were incompletely reported, these outcomes were not quantitatively pooled separately. When the seizure occurrence proportion was not directly reported, it was calculated by dividing the number of recipients with at least one seizure by the corresponding transplant cohort denominator.

Reported seizure-related clinical factors were also extracted and categorized as immunosuppressant-related neurotoxicity, metabolic or electrolyte disturbances, PRES, cerebrovascular, hypoxic–ischemic, or other structural brain lesions, infection or systemic complications, and pre-existing neurological conditions. Information not reported in the original studies was recorded as not reported (NR), and no values were imputed. Study authors were not contacted for additional information. No automation tools were used for data extraction.

### 2.5. Statistical Analysis

The primary outcome was the proportion of solid organ transplant recipients who experienced at least one seizure during the follow-up period reported in each study. This proportion was calculated by dividing the number of recipients who experienced one or more post-transplant seizures by the total number of transplant recipients at risk. Because follow-up duration varied across studies, the pooled estimate was interpreted as a summary of study-specific seizure occurrence proportions rather than as an incidence rate over a uniform observation period.

Study-specific proportions were logit-transformed and pooled using an inverse-variance random-effects model to account for anticipated clinical and methodological heterogeneity. Pooled estimates and their corresponding 95% confidence intervals (CIs) were subsequently back-transformed to the original proportion scale. Between-study variance (τ^2^) was estimated using the DerSimonian–Laird method, and Wald-type 95% CIs were calculated on the logit scale. No included study reported zero seizure cases; therefore, no continuity correction was applied. As a robustness analysis, the primary overall meta-analysis was additionally repeated using restricted maximum likelihood (REML) estimation of between-study variance with Hartung–Knapp adjustment of the confidence interval.

For analyses including at least five independent cohorts, 95% prediction intervals were calculated on the logit scale using a t distribution with k−2 degrees of freedom and subsequently back-transformed to the proportion scale. Prediction intervals were interpreted as estimates of the range within which the true seizure occurrence proportion might lie in a new population comparable to those included in the corresponding meta-analysis.

Statistical heterogeneity was evaluated using Cochran’s Q test, the *I*^2^ statistic, and τ^2^. Heterogeneity was interpreted in conjunction with differences in study design, transplant type, recipient characteristics, seizure ascertainment, and follow-up duration rather than solely according to fixed *I*^2^ thresholds. To further explore potential sources of between-study heterogeneity, exploratory univariable meta-regression analyses were performed using selected study-level characteristics as moderators. These included publication year, cohort sample size, follow-up/seizure ascertainment category, geographic region, recipient age-group classification, transplant type, and study design. All meta-regression models included the 32 independent cohorts and were fitted on the logit event-rate scale using random-effects meta-regression with the method-of-moments estimator for between-study variance (τ^2^). Regression coefficients and their 95% CIs were calculated using Z-distribution-based inference, with two-sided Z tests used for individual regression coefficients; omnibus Q tests were used for categorical moderators. Because cohort size was markedly skewed, the natural logarithm of sample size was used as the primary sample-size moderator, with raw sample size examined as a sensitivity analysis. For categorical moderators, the reference categories were early/index-hospitalization for follow-up/seizure ascertainment, Americas for geographic region, pediatric for recipient age group, heart transplantation for transplant type, and retrospective for study design. Because only 32 independent cohorts were available and several categorical moderators contained sparsely represented categories, multivariable meta-regression was not performed to avoid overfitting and unstable estimates. All meta-regression analyses were considered exploratory and were interpreted as study-level rather than patient-level associations.

Follow-up classification was based on the ascertainment window for the seizure outcome included in the quantitative analysis rather than on the overall clinical follow-up duration reported for other outcomes. Studies were classified as early/index-hospitalization when seizure ascertainment was restricted to the first 30 days after transplantation or to the index transplant hospitalization without explicit ascertainment of later post-discharge seizures; extended/long-term when seizure ascertainment explicitly continued beyond 30 days and included later post-transplant follow-up; and unclear/variable when the seizure ascertainment window could not be reliably determined. Studies restricted to the index hospitalization remained in the early/index-hospitalization category regardless of the actual duration of hospitalization unless later post-discharge seizure ascertainment was explicitly reported.

Subgroup analyses were conducted according to transplanted organ type and recipient age group. Additional subgroup analyses were performed according to follow-up/seizure ascertainment category. Differences between subgroup estimates were assessed using mixed-effects models with separate estimates of between-study variance across subgroups. Organ-specific analyses were performed separately for liver, kidney, heart, and lung transplant cohorts. Studies involving mixed organ populations were excluded from formal organ-specific comparisons unless separate organ-specific numerator and denominator data were available. Age-specific analyses were conducted separately for adult and pediatric cohorts, with mixed-age or insufficiently specified cohorts excluded from the formal adult-versus-pediatric comparison. Given the predominance of liver-only cohorts, age-stratified analyses were additionally performed within liver transplant cohorts. Detailed subgroup definitions, cohort-level follow-up/seizure ascertainment classifications, meta-regression specifications, and robustness analyses are provided in Supplementary Table S4.

Reported seizure-related clinical factors were categorized as immunosuppressant-related neurotoxicity, metabolic or electrolyte disturbances, PRES, cerebrovascular, hypoxic–ischemic, or other structural brain lesions, infection or systemic complications, and pre-existing neurological conditions. Because definitions, ascertainment methods, and reported measures varied substantially across studies, these factors were synthesized descriptively rather than quantitatively pooled. Reported findings were classified according to the type of evidence provided in the original study as adjusted seizure-specific associations, unadjusted seizure-specific associations, descriptive seizure-specific findings, or findings derived from broader clinical-context or composite neurological outcomes. Each individual finding was linked to the corresponding study rather than to a shared group of references. No formal certainty grading was applied to these descriptive clinical-factor findings. Categories were not mutually exclusive, and a study could contribute information to more than one category.

Sensitivity analyses were conducted by excluding studies primarily focused on PRES, excluding all cohorts selected on the basis of seizures or PRES, excluding the study judged to have a high risk of bias, restricting the analysis to studies judged to have a low risk of bias, and sequentially omitting each study in a leave-one-out analysis. Seizure- or PRES-focused studies were eligible for the primary analysis only when seizure occurrence could be calculated using a clearly defined underlying transplant cohort as the denominator.

Small-study effects were assessed for the primary overall meta-analysis using visual inspection of the funnel plot, Egger’s regression test, the Begg–Mazumdar rank correlation test, and the Duval and Tweedie trim-and-fill method. Because funnel plot asymmetry may arise from sources other than publication bias, particularly in the presence of substantial heterogeneity, these analyses were interpreted cautiously.

No formal assessment of the certainty of the body of evidence (e.g., GRADE) was performed because the primary objective was to synthesize observational estimates of seizure occurrence rather than to evaluate the comparative effects of an intervention.

Study characteristics and synthesis results were summarized in tables, and study-specific and pooled seizure occurrence proportions were displayed using forest plots.

All statistical tests were two-sided, and *p* values < 0.05 were considered statistically significant. Quantitative analyses were performed using Comprehensive Meta-Analysis version 4 (Biostat, Englewood, NJ, USA).

## 3. Results

### 3.1. Literature Search and Study Selection

**Figure 1:**
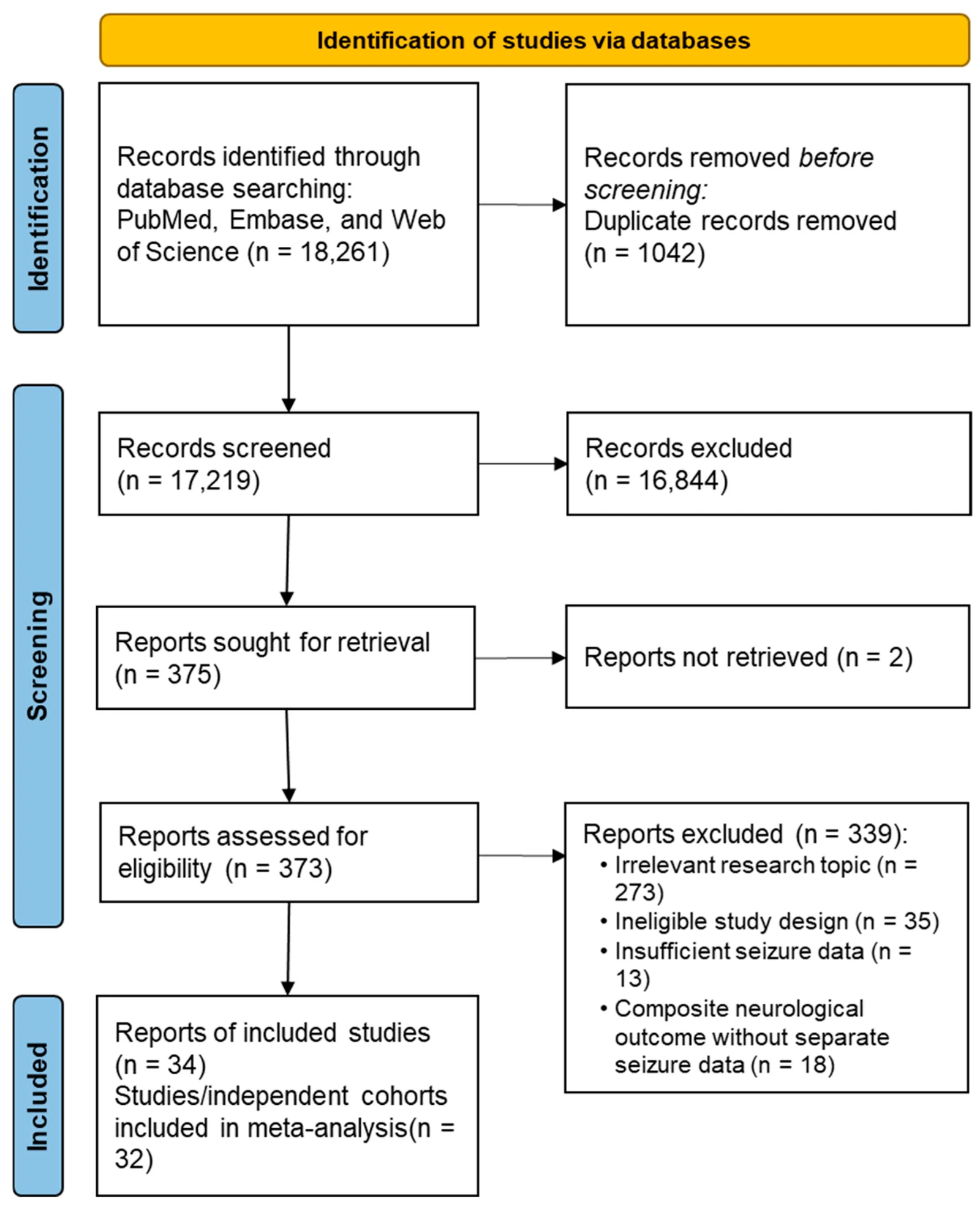
PRISMA 2020 flow diagram of study selection.

The database search identified 18,261 records (Figure 1). After 1042 duplicate records were removed, 17,219 records underwent title and abstract screening, of which 16,844 were excluded. The full texts of 375 reports were sought for retrieval, of which two could not be retrieved. Accordingly, 373 reports were assessed for eligibility. Of these, 339 were excluded because of an irrelevant research topic (*n* = 273), an ineligible study design (*n* = 35), insufficient data to calculate the seizure occurrence proportion (*n* = 13), or reporting of composite neurological complications without separate seizure-specific data (*n* = 18). A total of 34 reports met the eligibility criteria and were included in the systematic review [12,16,26,27,28,29,30,31,32,33,34,35,36,37,38,39,40,41,42,43,44,45,46,47,48,49,50,51,52,53,54,55,56,57]. After accounting for reports describing the same or substantially overlapping study populations, these reports represented 32 independent cohorts, all of which were included in the quantitative synthesis [12,16,26,27,28,29,30,31,32,33,34,35,36,37,38,39,40,41,42,43,44,45,46,47,48,49,50,51,52,53,54,55]. Two additional reports, Erol et al. (2008) [56] and Saner et al. (2007) [57], were not entered separately into the meta-analysis because their study populations overlapped with those reported by Erol et al. (2007) [37] and Saner et al. (2006) [33], respectively. The latter reports were retained for the quantitative analyses because they provided the larger or more complete cohorts. Selected reports that could reasonably appear eligible but were excluded after full-text assessment, together with the reasons for exclusion, are provided in Supplementary Table S3.

### 3.2. General Characteristics of the Included Studies

The quantitative synthesis included 32 independent cohorts comprising 8816 solid organ transplant recipients (Table 1). The studies were published between 2006 and 2025 and were conducted across 14 countries. Most used retrospective cohort or other retrospective observational designs, whereas only a small number were prospective. The median cohort size was 157.5 recipients, with individual cohort sizes ranging from 27 to 1265.

Liver transplantation constituted the largest organ-specific evidence base, with 21 liver-only cohorts. Heart, kidney, and lung transplantation were each represented by three cohorts. One additional cohort included pediatric liver and combined liver–bowel transplant recipients, and another included a mixed pediatric solid organ transplant population (Table 1). Based on the age-group classifications used in the analyses, 13 cohorts included adult recipients, 10 included pediatric recipients, and nine included mixed or insufficiently specified age groups.

Across the 32 independent cohorts, 380 of 8816 transplant recipients experienced at least one post-transplant seizure, corresponding to a crude seizure occurrence proportion of 4.3%. Among the 21 liver-only cohorts, 295 seizures occurred among 6817 recipients. Kidney, heart, and lung transplant cohorts contributed eight seizures among 512 recipients, 20 among 396 recipients, and 28 among 824 recipients, respectively. The two mixed-organ cohorts contributed 29 seizures among 267 recipients (Table 1).

Organ-specific analyses were restricted to cohorts in which a single transplanted organ could be identified. Accordingly, the liver-specific analysis included the 21 liver-only cohorts, whereas the pediatric liver/combined liver–bowel cohort and the mixed pediatric solid organ transplant cohort were included in the overall and pediatric age-group analyses but excluded from organ-specific analyses. The resulting organ-specific estimates are presented in Table 2 and Supplementary Figure S1, and the adult, pediatric, and liver age-stratified estimates are shown in Table 2.

### 3.3. Results of Risk-of-Bias Assessment

Risk of bias was assessed using the Joanna Briggs Institute critical appraisal checklist for studies reporting prevalence data. Using the exploratory review-specific operational classification described above, 22 of the 34 included reports were grouped as low risk, 11 as moderate risk, and one as high risk. Among the 32 reports selected to represent independent cohorts in the quantitative synthesis, 20 were grouped as low risk, 11 as moderate risk, and one as high risk. Detailed item-level judgments, which constituted the primary risk-of-bias assessment, are presented in Supplementary Table S1.

Most reports were judged to have an appropriate sampling frame and participant selection method and to provide an adequate description of the study population and setting. Statistical analyses were also generally appropriate. The most frequent concerns involved incomplete reporting of seizure ascertainment, particularly whether seizures were identified using valid and consistently applied methods. Other concerns included small sample sizes, insufficient information on population coverage or follow-up completeness, and the inclusion of selected populations, such as recipients with PRES or cohorts assembled specifically to investigate seizures. These features may have reduced the generalizability of the reported seizure occurrence proportions.

### 3.4. Overall Pooled Seizure Occurrence Proportion

**Figure 2:**
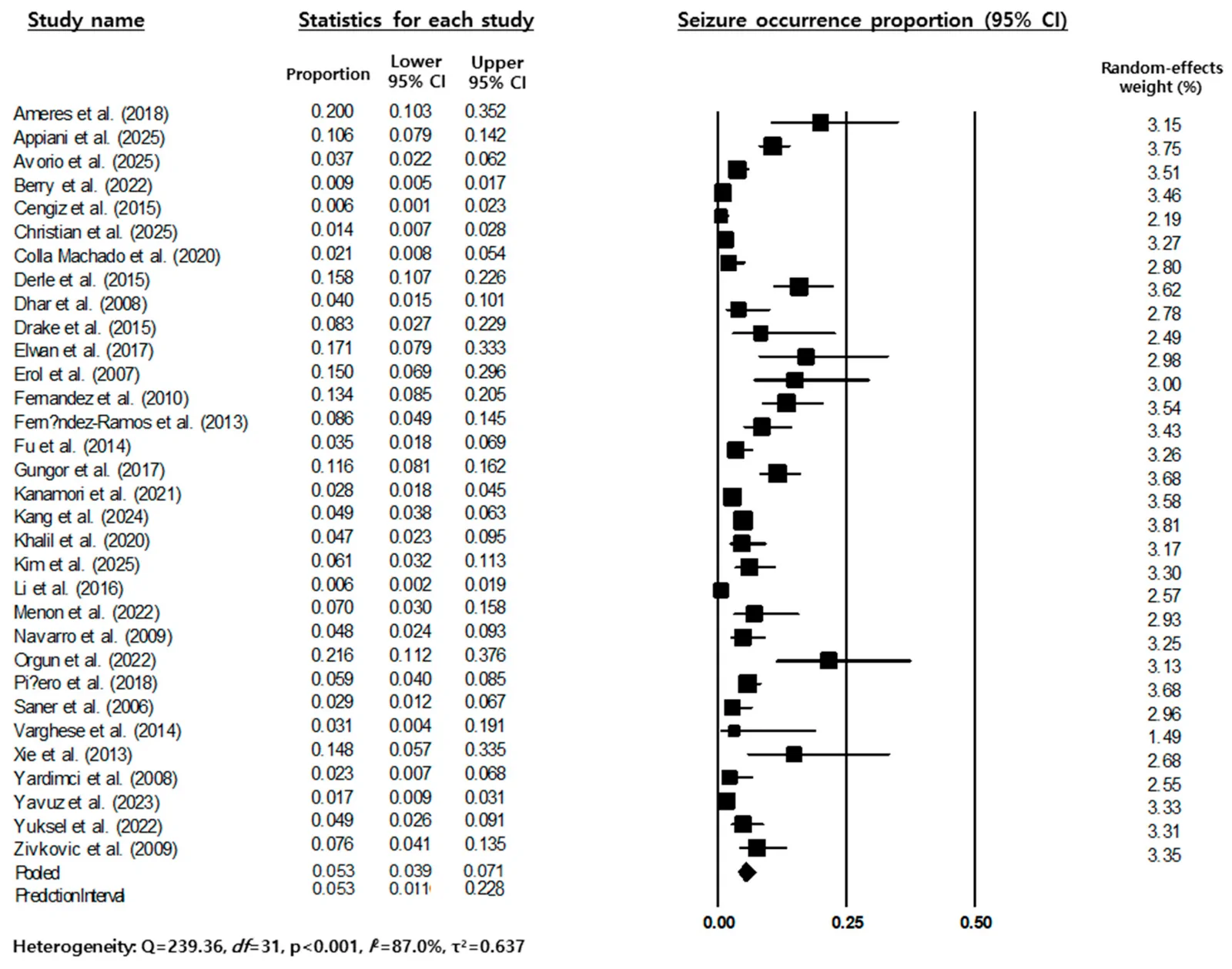
Forest plot of seizure occurrence proportions after solid organ transplantation. Individual studies are identified by the first author’s surname and publication year [12,16,26,27,28,29,30,31,32,33,34,35,36,37,38,39,40,41,42,43,44,45,46,47,48,49,50,51,52,53,54,55]. Random-effects model of logit-transformed proportion; between-study variance estimated using the DerSimonian-Laird method. Square sizes are proportional to random-effects weight; horizontal lines indicate 95% confidence intervals. The diamond represents the pooled estimate, and the horizontal bar below it represents the 95% prediction interval.

Across the 32 independent cohorts, study-specific seizure occurrence proportions ranged from 0.6% to 21.6%. Under a random-effects model, the pooled seizure occurrence proportion after solid organ transplantation was 5.3% (95% CI, 3.9–7.1%; Table 2 and Figure 2). Substantial between-study heterogeneity was observed (Q = 239.36, *df* = 31, *p* < 0.001; *I*^2^ = 87.0%; τ^2^ = 0.637). The 95% prediction interval ranged from 1.1% to 22.8%, indicating substantial variation in the underlying seizure occurrence proportion across transplant populations and clinical settings.

In the robustness analysis using REML estimation with Hartung–Knapp adjustment, the pooled estimate was 5.3% (95% CI, 3.7–7.4%; τ^2^ = 0.828), closely consistent with the primary DerSimonian–Laird estimate.

### 3.5. Subgroup Analyses

In the organ-specific analyses, the pooled seizure occurrence proportion was 5.4% among liver transplant recipients (21 cohorts; 95% CI, 3.7–7.7%; *I*^2^ = 88.7%), 6.4% among heart transplant recipients (three cohorts; 95% CI, 1.6–22.0%; *I*^2^ = 88.9%), 2.3% among kidney transplant recipients (three cohorts; 95% CI, 0.5–9.6%; *I*^2^ = 77.2%), and 4.3% among lung transplant recipients (three cohorts; 95% CI, 1.7–10.6%; *I*^2^ = 84.7%) (Table 2 and Supplementary Figure S1). For the liver transplant analysis, the 95% prediction interval ranged from 1.0% to 24.1%. Estimates for heart, kidney, and lung transplantation should be interpreted cautiously because each was based on only three cohorts. However, the formal between-group comparison did not indicate a significant difference in seizure occurrence across liver, heart, kidney, and lung transplant cohorts (Q = 1.443, df = 3, *p* = 0.695).

**Figure 3:**
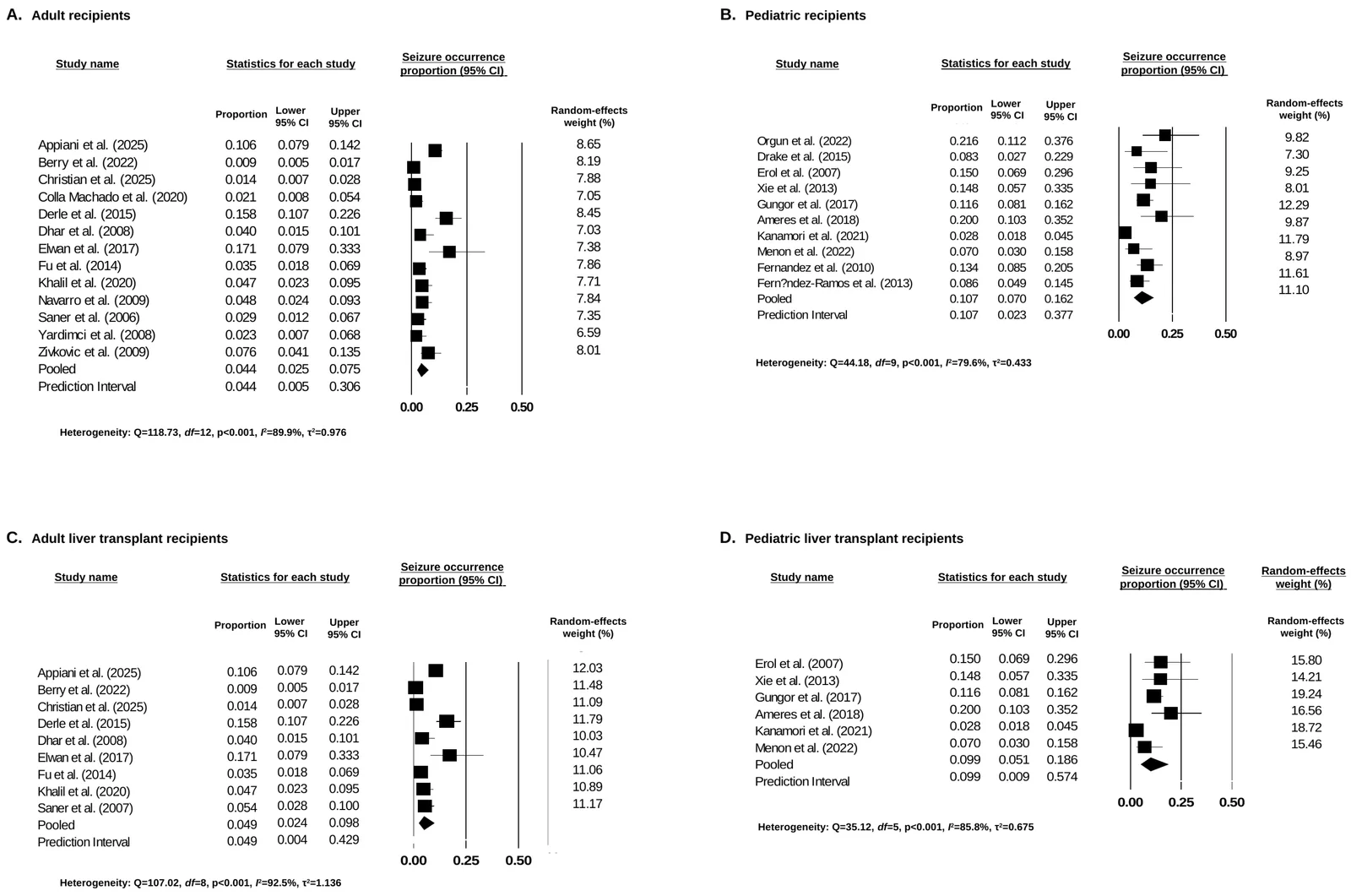
Forest plots of seizure occurrence proportions stratified by age group and transplant type: (**A**) adult recipients, (**B**) pediatric recipients, (**C**) adult liver transplant recipients, and (**D**) pediatric liver transplant recipients. Individual studies are identified by the first author’s surname and publication year [12,16,26,27,28,29,30,32,33,35,36,37,38,40,41,42,43,44,45,47,49,51,52,55]. Square sizes are proportional to random-effects weights, and horizontal lines represent 95% confidence intervals. Diamonds represent pooled estimates. The 95% prediction intervals are provided numerically below the pooled estimates.

In the age-specific analyses, the pooled seizure occurrence proportion was 4.4% among adult recipients (13 cohorts; 95% CI, 2.5–7.5%; *I*^2^ = 89.9%) and 10.7% among pediatric recipients (10 cohorts; 95% CI, 7.0–16.2%; *I*^2^ = 79.6%) (Table 2 and Figure 3A,B). The corresponding 95% prediction intervals were 0.5–30.6% and 2.3–37.7%, respectively. The pooled estimate was numerically higher in pediatric than in adult transplant populations. A formal mixed-effects subgroup comparison showed a significant difference between adult and pediatric cohorts (Q = 6.490, df = 1, *p* = 0.011).

Among liver-only cohorts, the pooled seizure occurrence proportion was 4.6% in adult recipients (nine cohorts; 95% CI, 2.2–9.4%; *I*^2^ = 92.7%) and 9.9% in pediatric recipients (six cohorts; 95% CI, 5.1–18.6%; *I*^2^ = 85.8%) (Table 2 and Figure 3C,D). The corresponding 95% prediction intervals were 0.4–42.9% and 0.9–57.4%, respectively. The pooled estimate was numerically higher in pediatric liver transplant recipients, although substantial heterogeneity remained in both age-specific liver transplant analyses. Within liver-only cohorts, the formal difference between adult and pediatric subgroups was not statistically significant (Q = 2.342, df = 1, *p* = 0.126).

In analyses stratified by follow-up/seizure ascertainment window, the pooled seizure occurrence proportion was 4.7% (95% CI, 2.7–8.3%; *I*^2^ = 87.3%) in early/index-hospitalization studies, 6.2% (95% CI, 4.2–9.1%; *I*^2^ = 82.0%) in extended/long-term studies, and 5.0% (95% CI, 2.5–9.7%; *I*^2^ = 91.2%) in studies with unclear or variable ascertainment periods. The between-group difference was not statistically significant (Q = 0.695, df = 2, *p* = 0.707). The corresponding subgroup results and cohort-level ascertainment classifications are provided in Supplementary Table S4.

### 3.6. Exploratory Meta-Regression Analyses

Exploratory univariable meta-regression analyses were performed to investigate potential study-level sources of heterogeneity. Cohort sample size was significantly associated with seizure occurrence. Using the natural logarithm of cohort size, larger cohorts were associated with lower seizure occurrence (β = −0.622, 95% CI, −0.881 to −0.363; *p* < 0.001), and cohort size explained approximately 31% of the between-study variance (R^2^ analog = 0.31), although substantial residual heterogeneity remained (*I*^2^ = 81.6%). An analysis using untransformed cohort size yielded a consistent association (β = −0.0016, 95% CI, −0.0025 to −0.0007; *p* < 0.001; R^2^ analog = 0.15).

Recipient age-group classification was also significantly associated with seizure occurrence (omnibus Q = 12.78, df = 2, *p* = 0.002; R^2^ analog = 0.07). With pediatric cohorts as the reference category, seizure occurrence was lower in adult cohorts (β = −0.964, 95% CI, −1.674 to −0.255; *p* = 0.008) and in mixed/insufficiently specified age cohorts (β = −1.367, 95% CI, −2.152 to −0.582; *p* < 0.001). In contrast, publication year (*p* = 0.342), follow-up/ascertainment category (*p* = 0.801), geographic region (*p* = 0.254), transplant type (*p* = 0.416), and study design (*p* = 0.435) were not significantly associated with seizure occurrence. Substantial residual heterogeneity remained across all models.

Detailed meta-regression model specifications and category-specific coefficients are provided in Supplementary Table S4.

### 3.7. Reported Seizure-Related Clinical Factors

Reported seizure-related clinical factors varied across studies and frequently overlapped within individual patients or cohorts (Table 3). The most commonly reported clinical contexts included immunosuppressant-related neurotoxicity, metabolic or electrolyte disturbances, posterior reversible encephalopathy syndrome (PRES), cerebrovascular, hypoxic–ischemic, or other structural brain lesions, infection or systemic complications, and pre-existing neurological conditions. In Table 3, each reported finding is linked to its corresponding study and classified as an adjusted seizure-specific association, an unadjusted seizure-specific association, a descriptive seizure-specific finding, or a clinical-context/composite finding. The term association is used only when a statistical relationship with a seizure-specific outcome was evaluated, whereas presumed etiologies reported descriptively are identified as descriptive findings. Because definitions and ascertainment methods differed substantially across studies and many reports provided descriptive or composite neurological outcomes rather than seizure-specific adjusted estimates, these factors were summarized qualitatively rather than quantitatively pooled. Additional robustness and selected-population sensitivity analyses are summarized in Supplementary Table S4.

### 3.8. Sensitivity Analyses

Sensitivity analyses produced pooled estimates similar to that of the primary analysis (Table 2 and Supplementary Figure S2). After excluding the two studies primarily focused on PRES, the pooled seizure occurrence proportion was 5.5% (30 cohorts; 95% CI, 4.1–7.4%; *I*^2^ = 87.0%), with a 95% prediction interval of 1.1–23.4% (Supplementary Figure S2A). Exclusion of the single study classified as having a high risk of bias yielded a pooled proportion of 5.6% (31 cohorts; 95% CI, 4.2–7.5%; *I*^2^ = 86.6%), with a 95% prediction interval of 1.2–22.9% (Supplementary Figure S2B).

When the analysis was restricted to the 20 independent cohorts classified as having a low risk of bias, the pooled seizure occurrence proportion was 5.8% (95% CI, 4.4–7.7%; *I*^2^ = 76.5%), with a 95% prediction interval of 1.8–17.4% (Supplementary Figure S2C). Although substantial heterogeneity remained, the *I*^2^ value was numerically lower than that observed in the primary analysis.

In the leave-one-out analysis, the pooled seizure occurrence proportion ranged from 5.1% to 5.7% after sequential omission of individual cohorts (Supplementary Figure S3). No single cohort materially changed the pooled estimate, suggesting that the primary result was not disproportionately influenced by any individual cohort.

An additional sensitivity analysis excluding all three seizure- or PRES-focused cohorts yielded a pooled seizure occurrence proportion of 5.3% (95% CI, 3.9–7.1%; *I*^2^ = 86.1%), with a 95% prediction interval of 1.1–21.9%. Thus, exclusion of cohorts with potentially enhanced neurological ascertainment did not materially alter the overall estimate.

### 3.9. Small-Study Effects

Visual inspection of the funnel plot showed no pronounced asymmetry (Supplementary Figure S4). Egger’s regression test did not indicate statistically significant funnel plot asymmetry (intercept = −1.341; two-sided *p* = 0.262), and the Begg–Mazumdar rank correlation test was also not statistically significant (Kendall’s τ = −0.137; two-sided *p* = 0.270). The Duval and Tweedie trim-and-fill method imputed no additional studies, and the adjusted pooled seizure occurrence proportion remained unchanged at 5.3% (95% CI, 3.9–7.1%). Overall, these analyses provided no clear evidence of small-study effects. However, the findings should be interpreted cautiously because funnel plot asymmetry tests may have limited interpretability in the presence of substantial between-study heterogeneity. The association with cohort size should not be equated with publication bias, as study size may also reflect differences in population selection and ascertainment intensity.

## 4. Discussion

In this systematic review and meta-analysis of 32 independent cohorts, the pooled seizure occurrence proportion after solid organ transplantation was 5.3% (95% CI, 3.9–7.1%). The wide 95% prediction interval of 1.1–22.8% indicates that seizure occurrence may vary substantially across transplant populations and clinical settings. Previous studies have reported widely varying proportions according to transplanted organ, recipient characteristics, follow-up duration, and seizure ascertainment methods [10,12,15,27,44,46,47,58,59,60,61]. Several were small, single-center studies focused on seizures, PRES, or broader neurological complications and thus represented populations in whom neurological events may have been more intensively detected [12,27,44,46,47]. Accordingly, the present estimate should be interpreted as an average of study-specific cumulative occurrence proportions rather than as a standardized incidence rate or a universal risk applicable to every transplant program. Because sufficiently standardized person-time denominators and observation windows were not available across studies, a pooled incidence rate could not be estimated reliably.

The substantial between-study heterogeneity is clinically plausible. Liver recipients may have pre-existing hepatic encephalopathy and marked perioperative metabolic or osmotic changes, heart recipients are particularly exposed to cerebrovascular injury, and lung recipients often require intensive immunosuppression associated with neurotoxicity and infection [12,27,44,46,47,58,59,60]. The etiologic spectrum may also differ according to post-transplant timing: perioperative hypoxic–ischemic injury, cerebrovascular events, metabolic disturbances, and CNI neurotoxicity are prominent early causes, whereas infection and other structural central nervous system disorders may become more relevant later [59,60,62,63]. However, the pooled outcome in the present review represents the occurrence of at least one post-transplant seizure and should not be interpreted as the incidence of epilepsy. The included studies did not provide sufficiently standardized information to allow separate quantitative synthesis of acute symptomatic versus unprovoked seizures, status epilepticus, or subsequent epilepsy. Heterogeneity remained substantial within organ-specific analyses, suggesting that organ type alone did not explain the observed variation. Exploratory meta-regression identified cohort size and age-group classification as study-level moderators of seizure occurrence. Larger cohorts tended to report lower seizure occurrence, with log-transformed cohort size explaining approximately 31% of the between-study variance. This association may reflect differences in cohort selection, seizure ascertainment, or other study-level characteristics and should not be interpreted as a patient-level effect of sample size. Age-group classification also showed a significant study-level association, with lower occurrence in adult than pediatric cohorts; however, this finding remains susceptible to ecological confounding by transplant type and other differences between the contributing studies and therefore does not establish pediatric age as an independent risk factor [64,65]. Perioperative metabolic disturbances and other transplant-related neurological complications may further contribute to variation in seizure occurrence across cohorts [66,67]. In contrast, publication year, follow-up/ascertainment category, geographic region, transplant type, and study design were not significant moderators, and substantial residual heterogeneity remained. Formal subgroup comparisons likewise showed no significant differences according to follow-up/ascertainment category (*p* = 0.707) or transplant organ type (*p* = 0.695). The primary pooled estimate was also essentially unchanged when REML estimation with Hartung–Knapp adjustment was used and when seizure- or PRES-focused cohorts were excluded, supporting the robustness of the summary estimate despite substantial residual heterogeneity.

Formal subgroup comparisons provided additional context for the age-related findings. The pooled seizure occurrence differed significantly between adult and pediatric cohorts overall (Q = 6.490, df = 1, *p* = 0.011), whereas the corresponding comparison restricted to liver-only cohorts was not statistically significant (Q = 2.342, df = 1, *p* = 0.126). Because the age groups were represented by different sets of studies and substantial residual heterogeneity remained, these study-level differences should not be interpreted as establishing pediatric age as an independent patient-level risk factor. The age-related findings therefore remain hypothesis-generating and require confirmation in studies directly comparing age groups.

The descriptive synthesis suggests that post-transplant seizures frequently arise from multiple overlapping clinical processes. These findings should not be interpreted uniformly as causal risk factors: some were supported by seizure-specific statistical associations, whereas others represented descriptive etiologic attribution, selected symptomatic populations, or associations with broader neurological outcomes. CNI–related neurotoxicity and PRES were repeatedly reported across transplant populations, and neurotoxicity was sometimes observed despite CNI concentrations within the therapeutic range [12,27,37,46,47,54,66,68,69]. Metabolic and osmotic disturbances were also prominent: abrupt reductions in sodium and serum osmolality preceded early seizures after pediatric kidney transplantation, while greater perioperative sodium changes were associated with neurological complications and seizure-specific outcomes after liver transplantation [16,40,51,66]. Cerebrovascular or hypoxic–ischemic injury, infection, and other structural brain lesions were also frequently reported clinical contexts [14,27,46,67,70,71]. A post-transplant seizure should therefore prompt evaluation of blood pressure, calcineurin inhibitor exposure and interacting medications, electrolyte and glucose abnormalities, renal and graft function, systemic or central nervous system infection, and cerebrovascular or structural lesions. Prognosis may depend more on the underlying disorder than on the seizure itself, with poorer outcomes reported when seizures accompany cerebrovascular disease, sepsis, rejection, or severe metabolic disturbance [14,51,67,70,71].

Antiseizure treatment in transplant recipients is complicated by potential pharmacokinetic interactions with immunosuppressive therapy. Enzyme-inducing antiseizure medications may increase the metabolism of calcineurin inhibitors and other immunosuppressants, potentially reducing immunosuppressant exposure and necessitating close therapeutic drug monitoring [61]. Agents with limited hepatic enzyme induction and lower interaction potential, such as levetiracetam, may therefore be preferable when clinically appropriate [61,72]. Selection of antiseizure therapy should nevertheless be individualized according to seizure type, hepatic and renal function, concomitant medications, and graft status. Evidence directly comparing antiseizure medications in transplant recipients remains limited, and the comparative superiority of any individual agent has not been established.

The pooled estimate was stable across the sensitivity analyses. Pooled proportions remained between 5.5% and 5.8% after excluding PRES-focused studies, excluding the high-risk-of-bias study, and restricting the analysis to low-risk-of-bias cohorts, while leave-one-out estimates ranged from 5.1% to 5.7%. Nevertheless, substantial heterogeneity persisted even in the low-risk-of-bias analysis, indicating that methodological restriction did not eliminate the underlying clinical diversity. Egger’s regression, Begg–Mazumdar rank correlation, and trim-and-fill analyses provided no clear evidence of small-study effects, and trim-and-fill did not alter the pooled estimate. These findings support the stability of the average estimate but should not be interpreted as proof that publication or selective-reporting bias was absent, particularly because funnel-plot–based methods have limited power in heterogeneous meta-analyses [73,74,75].

These findings highlight the need for prospective multicenter studies using standardized seizure definitions and prespecified ascertainment periods. Future studies should separately characterize seizure etiology and classification, including whether seizures were acute symptomatic or unprovoked, the occurrence of status epilepticus, seizure recurrence, and the subsequent development of epilepsy, recognizing that these characteristics may coexist. Electroencephalographic findings, neuroimaging abnormalities, and neurological outcomes should also be reported consistently [14,27,67,68,71]. Longitudinal assessment of CNI exposure, interacting medications, blood pressure, renal function, metabolic trajectories, graft function, and infection would permit more reliable evaluation of temporality and potentially modifiable pathways [16,40,51,54,66,68,69]. Seizure-specific adjusted analyses, formal organ- and age-interaction assessments, and long-term evaluation of graft loss, mortality, neurological disability, seizure recurrence, and subsequent epilepsy are also needed [14,51,67,70].

This study has several strengths. It quantitatively synthesized seizure-specific outcomes across the major solid organ transplant populations, distinguished independent cohorts from overlapping reports, and counted recipients experiencing at least one seizure rather than recurrent seizure episodes. It also incorporated prediction intervals, organ- and age-specific analyses, structured risk-of-bias assessment, and multiple sensitivity analyses. Several limitations should be acknowledged. Most studies were retrospective, single-center investigations, and seizure definitions, ascertainment methods, and observation periods were not standardized. Consequently, the pooled measure represents a study-specific occurrence proportion rather than an incidence rate over a common follow-up period. The evidence base was dominated by liver transplantation, with 21 of the 32 independent cohorts restricted to liver transplant recipients, whereas only three independent cohorts were available for each of heart, kidney, and lung transplantation. Consequently, the overall pooled estimate was substantially informed by the liver-transplant literature, and its generalizability to other solid organ transplant populations remains limited. Adult and pediatric estimates were derived from different sets of studies, and although a formal subgroup comparison showed a significant study-level difference, this finding should not be interpreted as establishing pediatric age as an independent patient-level risk factor. In addition, reported clinical factors were frequently based on small samples, selected symptomatic populations, unadjusted analyses, or composite neurological outcomes rather than seizure-specific adjusted models [12,16,27,31,34,37,40,44,46,47,51,54,66,68,69]. Although the consistency of the sensitivity analyses supports the stability of the pooled estimate, substantial residual heterogeneity limits its direct application as a uniform risk estimate for individual patients or transplant centers. The review process also had several limitations. Although no language restrictions were applied, the electronic literature search was limited to PubMed, Embase, and Web of Science, without a separate gray-literature search or backward or forward citation searching; therefore, relevant studies may have been missed. Study authors were not contacted for missing or unclear information. In addition, the review was not registered and no separate review protocol was prepared, limiting external verification of the review methods. No formal certainty-of-evidence assessment was performed.

## 5. Conclusions

The pooled study-specific seizure occurrence proportion after solid organ transplantation was 5.3%, but substantial variation across populations and ascertainment periods was reflected by the wide 95% prediction interval of 1.1–22.8%. The overall estimate was substantially informed by liver-transplant cohorts and should not be interpreted as a uniform individual risk across all transplant populations. The higher pooled occurrence observed in pediatric cohorts in the study-level subgroup analysis should be interpreted cautiously and does not establish pediatric age as an independent risk factor. Reported seizure-related clinical factors included CNI neurotoxicity, metabolic or osmotic disturbances, PRES, infection, and cerebrovascular or structural brain injury, but these findings do not uniformly represent seizure-specific causal risk factors. A post-transplant seizure should prompt timely evaluation for reversible and treatment-relevant causes, while standardized prospective studies are needed to clarify organ- and age-specific occurrence and long-term neurological, graft, and patient outcomes.

## Figures and Tables

**Table 1:** Characteristics of the included independent cohorts.

Study	Country	Study Period	Organ	Population	Study Design	Follow-Up/Seizure Ascertainment Window	Total N	Male, *n* (%)	Age	Seizures, *n*	Risk of Bias (JBI Score)
Ameres et al. [32]	Germany	2008–2013	Liver	Pediatric infants aged < 24 months	Retrospective cohort	Follow-up not clearly reported; 3-year mortality reported	40	22 (55.0)	Median 8.4 months (range 19 days–22 months)	8	Low (8/9)
Appiani et al. [53]	Argentina	Jan 2009–Jan 2019	Liver	Adult liver transplant recipients	Retrospective observational study; risk model	Within 30 days after transplantation	376	41% (*n* NR)	Median 54.8 years	40	Low (9/9)
Avorio et al. [52]	Italy	Jun 2014–Oct 2021	Liver	Orthotopic liver transplant recipients	Single-center retrospective study	During post-transplant hospitalization; early-onset seizures	376	NR	NR	14	Low (8/9)
Berry et al. [40]	USA	Jun 2012–Dec 2020	Liver	Adult liver transplant recipients	Retrospective cohort using an institutional registry	Same transplant hospitalization	1265	812 (64.2)	Median 59 years	12	Moderate (5/9)
Cengiz et al. [49]	Turkey	Jan 2005–Jul 2014	Kidney	Kidney transplant recipients	Retrospective clinical study	Post-transplant period; exact ascertainment window NR	344	NR	NR	2	Moderate (5/9)
Christian et al. [55]	India	Jan 2019–Sep 2022	Liver	Adult living-donor liver transplant recipients	Single-center cohort; predictive model	Neurological complications assessed 7–60 days after transplantation	569	455 (80.0)	Median 52 years	8	Low (8/9)
Colla Machado et al. [26]	Argentina	Nov 2014–Aug 2018	Heart	Adult heart transplant recipients	Retrospective single-center cohort	First postoperative week/in-hospital	193	143 (74.1)	Mean 51 ± 12 years	4	Low (7/9)
Derle et al. [12]	Turkey	2005–2013	Liver	Adult liver transplant recipients	Retrospective seizure-focused cohort	Post-transplant period; seizure onset variable	146	NR	NR	23	Moderate (5/9)
Dhar et al. [41]	Canada	Sep 2000–Mar 2002	Liver	Adult liver transplant recipients	Retrospective cohort	Within 30 days after transplantation	101	64 (63.4)	Median 50 years	4	Low (7/9)
Drake et al. [16]	USA	Jan 2013–Jan 2014	Kidney	Pediatric kidney transplant recipients	Retrospective case–control study within a cohort	Within 24 h after transplantation	36	NR	NR	3	Low (7/9)
Elwan et al. [39]	Egypt	Apr 2013–May 2014	Liver	Adult liver transplant recipients	Prospective observational cohort	Three months after transplantation	35	20 (57.1)	Mean 41.1 ± 8.0 years	6	Low (7/9)
Erol et al. [37]	Turkey	Sep 2001–Sep 2006	Liver	Pediatric liver transplant recipients	Retrospective single-center cohort	First 30 days emphasized	40	12 (30.0)	Median 13 years (range 2–18)	6	Low (8/9)
Fernandez et al. [45]	UK	2003–2007	Liver/liver–bowel	Pediatric liver and combined liver–small bowel transplant episodes	Single-center retrospective audit	Post-transplant episodes during the 4-year study period	127	54 (42.5)	Mean 5.47 years	17	Moderate (6/9)
Fernández-Ramos et al. [47]	Spain	2000–2011	Mixed solid organ	Pediatric heart, liver, and lung transplant recipients	Retrospective descriptive study	Postoperative period	140	NR	NR	12	Moderate (5/9)
Fu et al. [42]	USA	2003–2006	Liver	Adult liver transplant recipients	Retrospective review	First postoperative month	227	142 (62.6)	Mean 52 years	8	Low (7/9)
Gungor et al. [30]	Turkey	May 2006–June 2015	Liver	Pediatric liver transplant recipients	Retrospective cohort	Early and late events; late defined as ≥1 month	242	124 (51.2)	Mean 7.03 ± 5.47 years	28	Moderate (6/9)
Kanamori et al. [34]	Japan	Jan 2001–Jan 2020	Liver	Pediatric living-donor liver transplant recipients	Retrospective observational cohort	Post-transplant period; exact ascertainment window NR	602	NR	Median 1 year 1 month	17	Moderate (5/9)
Kang et al. [51]	Republic of Korea	Jul 2005–Dec 2021	Liver	Liver transplant recipients	Retrospective cohort with nested case–control analysis	Within 1 year after transplantation; median onset 11 days	1243	NR	NR	61	Low (9/9)
Khalil et al. [36]	Egypt	Nov 2011–Dec 2013	Liver	Adult living-donor liver transplant recipients	Prospective observational ICU cohort	One month after transplantation	149	135 (90.6)	Mean 50.16 ± 8.38 years	7	Low (8/9)
Kim et al. [54]	Republic of Korea	Feb 2013–Dec 2023	Lung	Lung transplant recipients	Retrospective PRES-focused cohort	Median PRES onset 15 days after transplantation	147	NR	NR for the full cohort	9	Low (7/9)
Li et al. [29]	China	Apr 2002–Sep 2013	Liver	Adult liver transplant recipients	Retrospective cohort	Postoperative period	474	394 (83.1)	Mean 48.2 years (range 19–79)	3	High (4/9)
Menon et al. [35]	India	Oct 2018–Nov 2019	Liver	Pediatric liver transplant recipients	Retrospective cohort	Median follow-up 15.5 months	71	35 (49.3)	NR	5	Low (7/9)
Navarro et al. [27]	France	3-year period	Heart	Adult heart transplant recipients	Retrospective cohort	Post-transplant period; mean seizure onset 30 days	166	129 (77.7)	Mean 44.2 ± 14.1 years	8	Low (8/9)
Orgun et al. [28]	Turkey	2007–2017	Heart	Pediatric heart transplant recipients	Retrospective single-center cohort	Post-transplant period; median onset 3 days	37	16 (43.2)	Median 13 years (range 2–18)	8	Moderate (5/9)
Piñero et al. [31]	Argentina	Oct 2001–Dec 2013	Liver	Adult and pediatric liver transplant recipients	Single-center cohort	Through discharge and outpatient follow-up; 5-year survival assessed	443	178 (40.2)	Adults: median 53 years (IQR 42–60); children: median 2 years (IQR 1–10)	26	Low (7/9)
Saner et al. [33]	Germany	Apr 2001–Mar 2004	Liver	Adult liver transplant ICU recipients	Retrospective cohort	ICU/postoperative period	174	NR	Mean 51 ± 16.6 years	5	Low (7/9)
Varghese et al. [43]	India	2009–2012	Liver	Liver transplant recipients receiving tacrolimus	Retrospective adverse-effect/TDM study	Up to 6 months after transplantation	32	29 (90.6)	Mean 44.3 ± 12.4 years	1	Moderate (6/9)
Xie et al. [44]	China	Jan 2007–Dec 2010	Liver	Pediatric liver transplant recipients	Single-center retrospective cohort	Seizure ascertainment: first 2 weeks; overall cohort follow-up: mean 33.7 months	27	7 (25.9)	Mean 3.36 ± 3.9 years	4	Low (8/9)
Yardimci et al. [48]	Turkey	Jan 2005–Dec 2007	Kidney	Adult kidney transplant recipients	Retrospective cohort	Mean follow-up 17.3 months	132	97 (73.5)	Mean 34.32 ± 0.90 years	3	Low (8/9)
Yavuz et al. [50]	Germany	Jan 2012–Dec 2019	Lung	Lung transplant recipients	Retrospective PRES-focused cohort with nested comparison	Post-transplant PRES; exact seizure ascertainment window NR	545	297 (54.5)	Mean 51.06 ± 12.42 years	9	Moderate (5/9)
Yuksel et al. [38]	Turkey	NR	Liver	Adult liver transplant recipients	Retrospective cohort	Preoperative, early postoperative, and late postoperative periods	185	60 (32.4)	Mean 47.22 ± 11.18 years	9	Moderate (6/9)
Zivkovic et al. [46]	USA	2001–2003	Lung	Adult lung allograft recipients	Retrospective cohort	Follow-up through 31 Dec 2005	132	69 (52.3)	Mean 49.57 ± 12.53 years	10	Low (7/9)

Abbreviations: ICU, intensive care unit; IQR, interquartile range; JBI, Joanna Briggs Institute; NR, not reported; PRES, posterior reversible encephalopathy syndrome; TDM, therapeutic drug monitoring.

**Table 2:** Summary of meta-analysis results.

Analysis	No. of Cohorts	Pooled Proportion (%)	95% CI (%)	*I^2^* (%)	95% PI (%)
Primary analysis					
Overall	32	5.3	3.9–7.1	87.0	1.1–22.8
Organ-specific subgroup analyses					
Liver	21	5.4	3.7–7.7	88.7	1.0–24.1
Heart	3	6.4	1.6–22.0	88.9	—
Kidney	3	2.3	0.5–9.6	77.2	—
Lung	3	4.3	1.7–10.6	84.7	—
Age-specific subgroup analyses					
Adult	13	4.4	2.5–7.5	89.9	0.5–30.6
Pediatric	10	10.7	7.0–16.2	79.6	2.3–37.7
Age-specific analyses restricted to liver transplantation					
Adult liver	9	4.6	2.2–9.4	92.7	0.4–42.9
Pediatric liver	6	9.9	5.1–18.6	85.8	0.9–57.4
Sensitivity analyses					
Excluding PRES-focused studies	30	5.5	4.1–7.4	87.0	1.1–23.4
Excluding the high-risk-of-bias study	31	5.6	4.2–7.5	86.6	1.2–22.9
Low-risk-of-bias cohorts only	20	5.8	4.4–7.7	76.5	1.8–17.4
Excluding seizure- or PRES-focused studies	29	5.3	3.9–7.1	86.1	1.1–21.9

Abbreviations: CI, confidence interval; PI, prediction interval; PRES, posterior reversible encephalopathy syndrome. Prediction intervals were not presented for analyses including fewer than five cohorts. Pooled estimates were calculated using random-effects models with logit-transformed proportions.

**Table 3:** Summary of reported seizure-related clinical factors according to evidence type.

Clinical Factor Category	Individual Reported Finding	Evidence Classification	Study/Reference
Immunosuppressant-related neurotoxicity	Higher tacrolimus trough concentration remained significantly associated with post-transplant seizure after multivariable analysis in a pediatric liver-transplant cohort.	Adjusted seizure-specific association	Xie et al. (2014) [44]
	Immunosuppressive drug-related seizure was identified in 8 of 23 seizure cases (34.8%); drug blood levels were reported to be within the normal range in these cases.	Descriptive seizure-specific finding	Derle et al. (2015) [12]
	Immunosuppressive agents were considered the primary cause of 13 of 16 neurological-complication episodes in a pediatric liver-transplant cohort.	Descriptive clinical-context/composite finding	Erol et al. (2007) [37]
Metabolic or electrolyte disturbances	A perioperative delta sodium level ≥ 4 mmol/L remained significantly associated with post-transplant seizure after multivariable adjustment (OR 5.38, 95% CI 2.55–12.3).	Adjusted seizure-specific association	Kang et al. (2024) [51]
	Children with seizures had greater acute decreases in serum sodium and calculated serum osmolality than controls (*p* < 0.001).	Unadjusted seizure-specific association	Drake et al. (2015) [16]
	Metabolic imbalance was identified as the presumed underlying condition in 4 of 23 seizure cases (17.4%).	Descriptive seizure-specific finding	Derle et al. (2015) [12]
	Each 5 mmol/L increase in perioperative delta sodium was associated with 47% greater adjusted odds of a post-transplant central nervous system complication.	Adjusted clinical-context/composite association	Berry et al. (2022) [40]
	Perioperative delta sodium and post-transplant hypomagnesemia were reported as independent risk factors for broader neurological events in adult liver-transplant recipients.	Adjusted clinical-context/composite association	Piñero et al. (2018) [31]
Posterior reversible encephalopathy syndrome (PRES)	PRES attributed to cyclosporine treatment was identified in 4 of 8 heart-transplant recipients with post-transplant seizures.	Descriptive seizure-specific finding	Navarro et al. (2010) [27]
	Among patients selected for PRES-like neurological symptoms after lung transplantation, seizures were more frequent in confirmed PRES than non-PRES cases (81.8% vs. 21.1%; *p* = 0.002).	Unadjusted clinical-context association in a selected PRES-focused population	Yavuz et al. (2023) [50]
	Seizures were the predominant clinical manifestation among lung-transplant recipients who developed PRES (9 of 11, 81.8%).	Descriptive PRES-focused clinical finding	Kim et al. (2025) [54]
Cerebrovascular, hypoxic–ischemic, or structural brain lesions	Cortical ischemic stroke was identified as an epileptogenic factor in 5 of 8 heart-transplant recipients with seizures.	Descriptive seizure-specific finding	Navarro et al. (2010) [27]
	Structural brain lesions were identified as the presumed underlying condition in 3 of 23 seizure cases (13.0%).	Descriptive seizure-specific finding	Derle et al. (2015) [12]
	Structural brain lesions were identified in 7 of 40 patients with early post-liver-transplant seizures.	Descriptive seizure-specific finding	Appiani et al. (2025) [53]
Infection or systemic complications	Sepsis was identified as the presumed underlying condition in 2 of 23 seizure cases (8.7%).	Descriptive seizure-specific finding	Derle et al. (2015) [12]
	Post-transplant infection was more frequent among patients with seizures than among those without seizures (62.5% vs. 33.4%; *p* = 0.001).	Unadjusted seizure-specific association	Appiani et al. (2025) [53]
	Preoperative infection remained independently associated with broader postoperative neurological complications (OR 2.83, 95% CI 1.47–5.44).	Adjusted clinical-context/composite association	Fu et al. (2014) [42]
	Opportunistic infections accounted for 11% of neurological complications after lung transplantation.	Descriptive clinical-context/composite finding	Živković et al. (2009) [46]
Pre-existing neurological conditions	Prior epilepsy (17.5% vs. 2.7%; *p* < 0.001) and active pre-transplant portosystemic encephalopathy (50.0% vs. 29.8%; *p* = 0.016) were more frequent among patients who developed post-transplant seizures.	Unadjusted seizure-specific associations	Appiani et al. (2025) [53]
	Pre-transplant hepatic encephalopathy remained the only significant predictor of broader post-transplant neurological complications after multivariable analysis in a pediatric liver-transplant cohort.	Adjusted clinical-context/composite association	Menon et al. (2022) [35]

Notes: Evidence classifications refer to the analytical basis reported in the original study. “Adjusted seizure-specific association” indicates a multivariable analysis with seizure as the outcome; “unadjusted seizure-specific association” indicates a crude, bivariate, or univariable comparison with a seizure-specific outcome; “descriptive seizure-specific finding” indicates descriptive attribution or characterization among seizure cases without an adjusted association estimate. Clinical-context/composite findings were based on broader neurological outcomes or selected clinical populations rather than a seizure-specific outcome. Categories are not mutually exclusive, and the summarized findings should not be interpreted as uniformly causal. Abbreviations: CI, confidence interval; OR, odds ratio; PRES, posterior reversible encephalopathy syndrome.

## Data Availability

All data used in this systematic review and meta-analysis were obtained from published studies cited in the article. The extracted and analyzed data and the standardized data extraction form supporting the findings are available from the corresponding author upon reasonable request. Detailed item-level risk-of-bias assessments and complete database search strategies are provided in Supplementary Tables S1 and S2, respectively. No custom analytic code was developed for this review.

## Supplementary Materials

The following supporting information can be downloaded at https://www.mdpi.com/article/10.3390/jcm15218354/s1: Figure S1: Organ-specific forest plots of seizure occurrence proportions after solid organ transplantation; Figure S2: Sensitivity analyses of the pooled seizure occurrence proportion after solid organ transplantation; Figure S3: Leave-one-out sensitivity analysis of the pooled seizure occurrence proportion after solid organ transplantation; Figure S4: Funnel plot of the 32 independent cohorts included in the primary meta-analysis of seizure occurrence after solid organ transplantation; Table S1: Detailed Joanna Briggs Institute risk-of-bias assessment of the included reports; Table S2: Complete search strategies for PubMed, Embase, and Web of Science; Table S3: Reports that appeared potentially eligible but were excluded after full-text assessment [76,77,78,79,80,81,82,83,84,85]; Table S4: Additional subgroup, meta-regression, robustness, and follow-up ascertainment analyses.

## Abbreviations

The following abbreviations are used in this manuscript:

CI	confidence interval
CNI	calcineurin inhibitor
HE	hepatic encephalopathy
ICU	intensive care unit
IQR	interquartile range
JBI	Joanna Briggs Institute
NR	not reported
PI	prediction interval
PRES	posterior reversible encephalopathy syndrome
PRISMA	Preferred Reporting Items for Systematic Reviews and Meta-Analyses
SOT	solid organ transplantation
TDM	therapeutic drug monitoring
