# Supplementary material for: Seizure Occurrence After Solid Organ Transplantation: A Systematic Review and Meta-Analysis
Source: J Clin Med. 2026 Oct 27;15(21):8354. doi: 10.3390/jcm15218354 (PMC13764164; doi:10.3390/jcm15218354)
Supplement: Supplementary file 1 [file jcm-15-08354-s001.zip › jcm-4537793-supplementary.pdf]

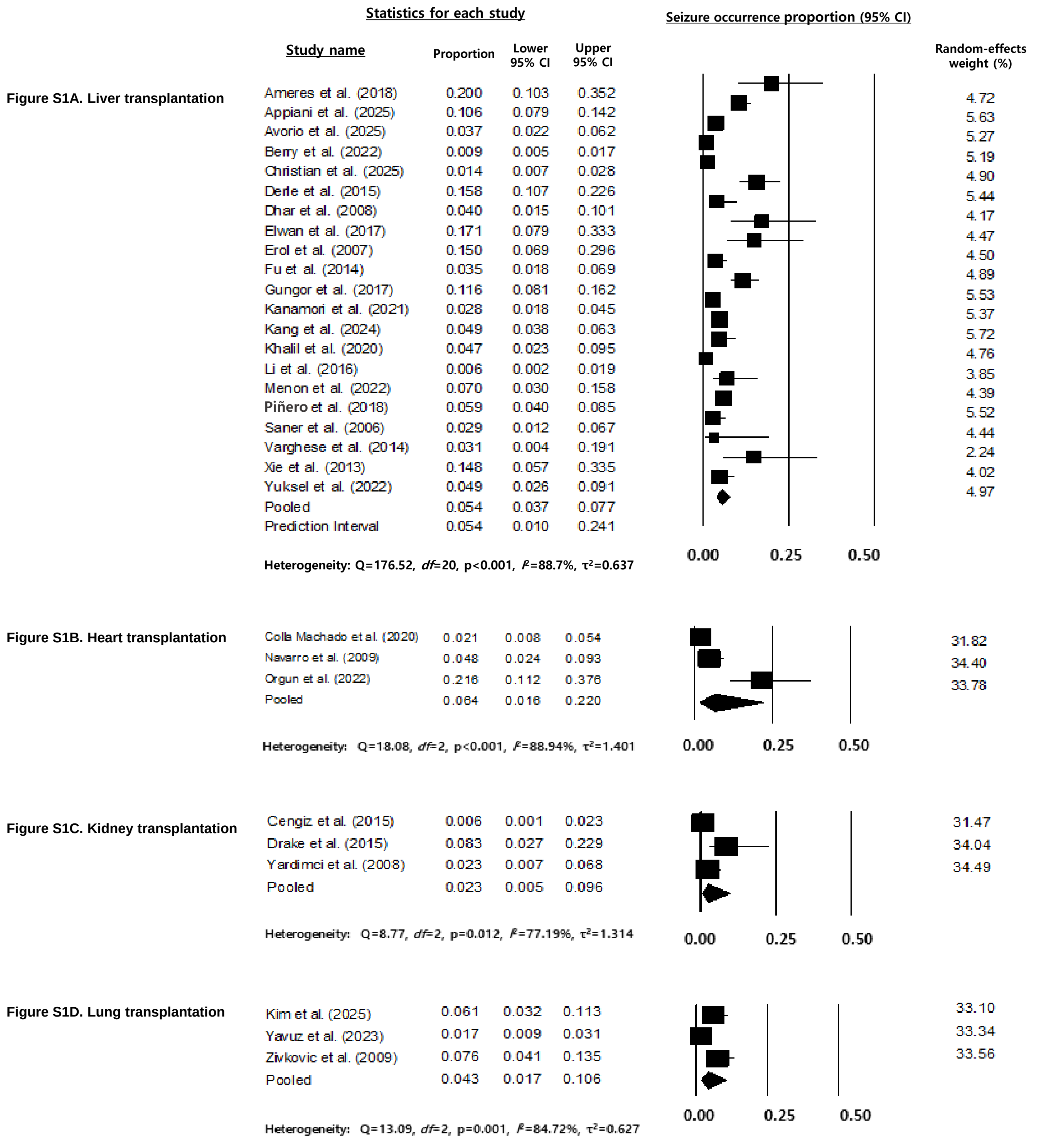


Figure S1. Organ-specific forest plots of seizure occurrence proportions after solid organ transplantation. (A) Liver transplantation; (B) heart transplantation; (C) kidney transplantation; and (D) lung transplantation. Squares represent study-specific seizure occurrence proportions, with square sizes proportional to study weights, and horizontal lines indicate 95% confidence intervals. Diamonds represent pooled estimates obtained using inverse-variance random-effects models. The 95% prediction interval is shown only for the liver transplantation analysis, which included at least five independent cohorts. The studies included in these analyses are cited in References [12,16,26–55].

**Seizure occurrence proportion (95% CI)**

| Study name | Proportion | Lower 95% CI | Upper 95% CI | Random-effects weight (%) |
|---|---|---|---|---|
| Ameres et al. (2018) | 0.200 | 0.103 | 0.352 | 3.37 |
| Appiani et al. (2025) | 0.106 | 0.079 | 0.142 | 4.03 |
| Avorio et al. (2025) | 0.037 | 0.022 | 0.062 | 3.76 |
| Berry et al. (2022) | 0.009 | 0.005 | 0.017 | 3.71 |
| Cengiz et al. (2015) | 0.006 | 0.001 | 0.023 | 2.34 |
| Christian et al. (2025) | 0.014 | 0.007 | 0.028 | 3.50 |
| Colla Machado et al. (2020) | 0.021 | 0.008 | 0.054 | 2.99 |
| Derle et al. (2015) | 0.158 | 0.107 | 0.226 | 3.89 |
| Dhar et al. (2008) | 0.040 | 0.015 | 0.101 | 2.97 |
| Drake et al. (2015) | 0.083 | 0.027 | 0.229 | 2.66 |
| Elwan et al. (2017) | 0.171 | 0.079 | 0.333 | 3.19 |
| Erol et al. (2007) | 0.150 | 0.069 | 0.296 | 3.21 |
| Fernandez et al. (2010) | 0.134 | 0.085 | 0.205 | 3.80 |
| **Fernández-Ramos** et al. (2013) | 0.086 | 0.049 | 0.145 | 3.67 |
| Fu et al. (2014) | 0.035 | 0.018 | 0.069 | 3.49 |
| Gungor et al. (2017) | 0.116 | 0.081 | 0.162 | 3.95 |
| Kanamori et al. (2021) | 0.028 | 0.018 | 0.045 | 3.84 |
| Kang et al. (2024) | 0.049 | 0.038 | 0.063 | 4.09 |
| Khalil et al. (2020) | 0.047 | 0.023 | 0.095 | 3.40 |
| Li et al. (2016) | 0.006 | 0.002 | 0.019 | 2.74 |
| Menon et al. (2022) | 0.070 | 0.030 | 0.158 | 3.13 |
| Navarro et al. (2009) | 0.048 | 0.024 | 0.093 | 3.48 |
| Orgun et al. (2022) | 0.216 | 0.112 | 0.376 | 3.35 |
| **Piñero** et al. (2018) | 0.059 | 0.040 | 0.085 | 3.95 |
| Saner et al. (2006) | 0.029 | 0.012 | 0.067 | 3.17 |
| Varghese et al. (2014) | 0.031 | 0.004 | 0.191 | 1.59 |
| Xie et al. (2013) | 0.148 | 0.057 | 0.335 | 2.87 |
| Yardimci et al. (2008) | 0.023 | 0.007 | 0.068 | 2.73 |
| Yuksel et al. (2022) | 0.049 | 0.026 | 0.091 | 3.55 |
| Zivkovic et al. (2009) | 0.076 | 0.041 | 0.135 | 3.59 |
| Pooled | 0.055 | 0.041 | 0.074 | |
| Prediction Interval | 0.055 | 0.011 | 0.234 | |

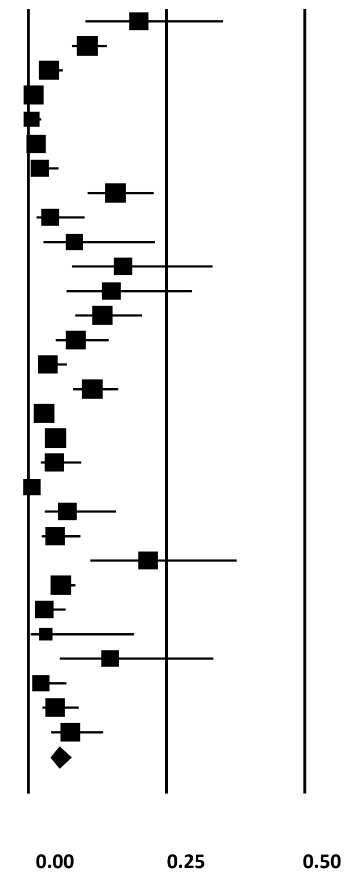


**Heterogeneity: Q=223.08, *df*=29, p<0.001, $I^2$=87.0%, $\tau^2$=0.628**

Figure S2A. Forest plot of the sensitivity analysis excluding two studies primarily focused on posterior reversible encephalopathy syndrome (PRES) [12,16,26–49,51–53,55].

| Study name | Proportion | Lower 95% CI | Upper 95% CI | Random-effects weight (%) |
|---|---|---|---|---|
| Ameres et al. (2018) | 0.200 | 0.103 | 0.352 | 3.22 |
| Appiani et al. (2025) | 0.106 | 0.079 | 0.142 | 3.89 |
| Avorio et al. (2025) | 0.037 | 0.022 | 0.062 | 3.62 |
| Berry et al. (2022) | 0.009 | 0.005 | 0.017 | 3.57 |
| Cengiz et al. (2015) | 0.006 | 0.001 | 0.023 | 2.21 |
| Christian et al. (2025) | 0.014 | 0.007 | 0.028 | 3.36 |
| Colla Machado et al. (2020) | 0.021 | 0.008 | 0.054 | 2.85 |
| Derle et al. (2015) | 0.158 | 0.107 | 0.226 | 3.75 |
| Dhar et al. (2008) | 0.040 | 0.015 | 0.101 | 2.83 |
| Drake et al. (2015) | 0.083 | 0.027 | 0.229 | 2.53 |
| Elwan et al. (2017) | 0.171 | 0.079 | 0.333 | 3.04 |
| Erol et al. (2007) | 0.150 | 0.069 | 0.296 | 3.06 |
| Fernandez et al. (2010) | 0.134 | 0.085 | 0.205 | 3.65 |
| Fernández-Ramos et al. (2013) | 0.086 | 0.049 | 0.145 | 3.53 |
| Fu et al. (2014) | 0.035 | 0.018 | 0.069 | 3.34 |
| Gungor et al. (2017) | 0.116 | 0.081 | 0.162 | 3.81 |
| Kanamori et al. (2021) | 0.028 | 0.018 | 0.045 | 3.69 |
| Kang et al. (2024) | 0.049 | 0.038 | 0.063 | 3.95 |
| Khalil et al. (2020) | 0.047 | 0.023 | 0.095 | 3.25 |
| Kim et al. (2025) | 0.061 | 0.032 | 0.113 | 3.40 |
| Menon et al. (2022) | 0.070 | 0.030 | 0.158 | 2.99 |
| Navarro et al. (2009) | 0.048 | 0.024 | 0.093 | 3.33 |
| Orgun et al. (2022) | 0.216 | 0.112 | 0.376 | 3.21 |
| Piñero et al. (2018) | 0.059 | 0.040 | 0.085 | 3.81 |
| Saner et al. (2006) | 0.029 | 0.012 | 0.067 | 3.02 |
| Varghese et al. (2014) | 0.031 | 0.004 | 0.191 | 1.49 |
| Xie et al. (2013) | 0.148 | 0.057 | 0.335 | 2.73 |
| Yardimci et al. (2008) | 0.023 | 0.007 | 0.068 | 2.59 |
| Yavuz et al. (2023) | 0.017 | 0.009 | 0.031 | 3.42 |
| Yuksel et al. (2022) | 0.049 | 0.026 | 0.091 | 3.40 |
| Zivkovic et al. (2009) | 0.076 | 0.041 | 0.135 | 3.44 |
| Pooled | 0.056 | 0.042 | 0.075 | |
| Prediction Interval | 0.056 | 0.012 | 0.229 | |

**Seizure occurrence proportion (95% CI)**

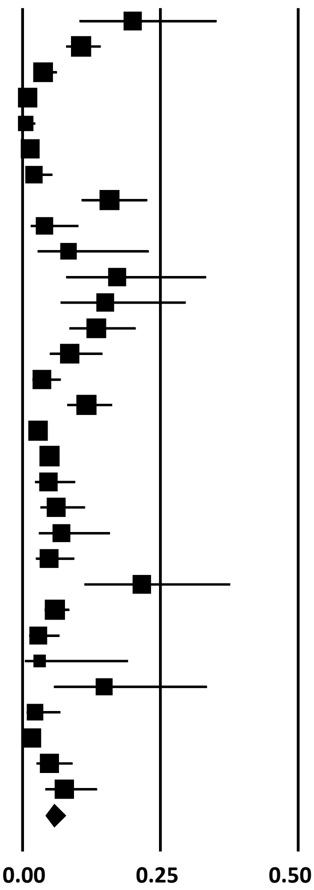


Heterogeneity: Q=233.34, *df*=30, $p<0.001$, $I^2$=86.6%, $\tau^2$=0.597

Figure S2B. Forest plot of the sensitivity analysis excluding the study classified as having a high risk of bias [12,16,26–28,30–55].

| Study name | Proportion | Lower 95% CI | Upper 95% CI | Random-effects weight (%) |
|---|---|---|---|---|
| Ameres et al. (2018) | 0.200 | 0.103 | 0.352 | 4.95 |
| Appiani et al. (2025) | 0.106 | 0.079 | 0.142 | 6.80 |
| Avorio et al. (2025) | 0.037 | 0.022 | 0.062 | 5.99 |
| Christian et al. (2025) | 0.014 | 0.007 | 0.028 | 5.28 |
| Colla Machado et al. (2020) | 0.021 | 0.008 | 0.054 | 4.10 |
| Dhar et al. (2008) | 0.040 | 0.015 | 0.101 | 4.06 |
| Drake et al. (2015) | 0.083 | 0.027 | 0.229 | 3.44 |
| Elwan et al. (2017) | 0.171 | 0.079 | 0.333 | 4.53 |
| Erol et al. (2007) | 0.150 | 0.069 | 0.296 | 4.57 |
| Fu et al. (2014) | 0.035 | 0.018 | 0.069 | 5.25 |
| Kang et al. (2024) | 0.049 | 0.038 | 0.063 | 7.02 |
| Khalil et al. (2020) | 0.047 | 0.023 | 0.095 | 5.02 |
| Kim et al. (2025) | 0.061 | 0.032 | 0.113 | 5.39 |
| Menon et al. (2022) | 0.070 | 0.030 | 0.158 | 4.41 |
| Navarro et al. (2009) | 0.048 | 0.024 | 0.093 | 5.23 |
| Piñero et al. (2018) | 0.059 | 0.040 | 0.085 | 6.55 |
| Saner et al. (2006) | 0.029 | 0.012 | 0.067 | 4.48 |
| Xie et al. (2013) | 0.148 | 0.057 | 0.335 | 3.84 |
| Yardimci et al. (2008) | 0.023 | 0.007 | 0.068 | 3.56 |
| Zivkovic et al. (2009) | 0.076 | 0.041 | 0.135 | 5.52 |
| Pooled | 0.058 | 0.044 | 0.077 | |
| Prediction Interval | 0.058 | 0.018 | 0.174 | |

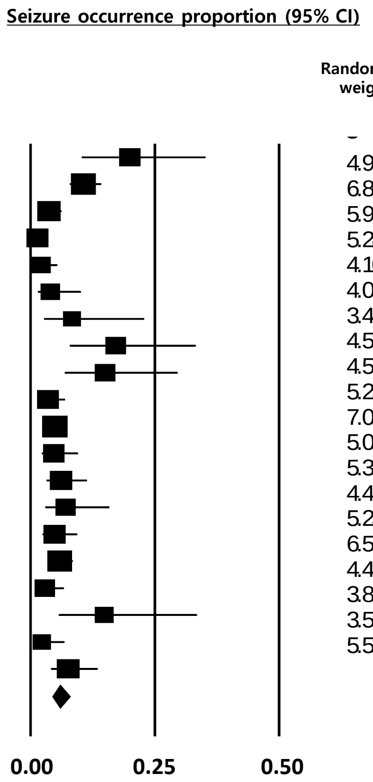


Heterogeneity: Q=80.79, *df*=19, $p<0.001$, $I^2$=76.48%, $\tau^2$=0.317

Figure S2C. Forest plot of the sensitivity analysis restricted to cohorts classified as having a low risk of bias [16,26,27,31–33,35–37,39,41,42,44,46,48,51–55].

Supplementary Figure S2. Sensitivity analyses of the pooled seizure occurrence proportion after solid organ transplantation.

| Study omitted | Seizure occurrence proportion (95% CI) | | |
|---|---|---|---|
| | Proportion | Lower 95% CI | Upper 95% CI |
| Ameres et al. (2018) | 0.051 | 0.038 | 0.068 |
| Appiani et al. (2025) | 0.052 | 0.038 | 0.070 |
| Avorio et al. (2025) | 0.054 | 0.040 | 0.072 |
| Berry et al. (2022) | 0.057 | 0.043 | 0.074 |
| Cengiz et al. (2015) | 0.056 | 0.042 | 0.074 |
| Christian et al. (2025) | 0.056 | 0.041 | 0.074 |
| Colla Machado et al. (2020) | 0.054 | 0.040 | 0.073 |
| Derle et al. (2015) | 0.051 | 0.038 | 0.068 |
| Dhar et al. (2008) | 0.053 | 0.040 | 0.072 |
| Drake et al. (2015) | 0.052 | 0.039 | 0.070 |
| Elwan et al. (2017) | 0.051 | 0.038 | 0.069 |
| Erol et al. (2007) | 0.051 | 0.038 | 0.069 |
| Fernandez et al. (2010) | 0.051 | 0.038 | 0.069 |
| **Fernández-Ramos** et al. (2013) | 0.052 | 0.038 | 0.070 |
| Fu et al. (2014) | 0.054 | 0.040 | 0.072 |
| Gungor et al. (2017) | 0.051 | 0.038 | 0.069 |
| Kanamori et al. (2021) | 0.054 | 0.040 | 0.073 |
| Kang et al. (2024) | 0.053 | 0.038 | 0.073 |
| Khalil et al. (2020) | 0.053 | 0.039 | 0.072 |
| Kim et al. (2025) | 0.053 | 0.039 | 0.071 |
| Li et al. (2016) | 0.056 | 0.042 | 0.075 |
| Menon et al. (2022) | 0.053 | 0.039 | 0.071 |
| Navarro et al. (2009) | 0.053 | 0.039 | 0.072 |
| Orgun et al. (2022) | 0.051 | 0.038 | 0.068 |
| **Piñero** et al. (2018) | 0.053 | 0.039 | 0.072 |
| Saner et al. (2006) | 0.054 | 0.040 | 0.073 |
| Varghese et al. (2014) | 0.053 | 0.040 | 0.072 |
| Xie et al. (2013) | 0.051 | 0.038 | 0.069 |
| Yardimci et al. (2008) | 0.054 | 0.040 | 0.073 |
| Yavuz et al. (2023) | 0.055 | 0.041 | 0.074 |
| Yuksel et al. (2022) | 0.053 | 0.039 | 0.072 |
| Zivkovic et al. (2009) | 0.052 | 0.039 | 0.071 |
| Pooled | 0.053 | 0.039 | 0.071 |

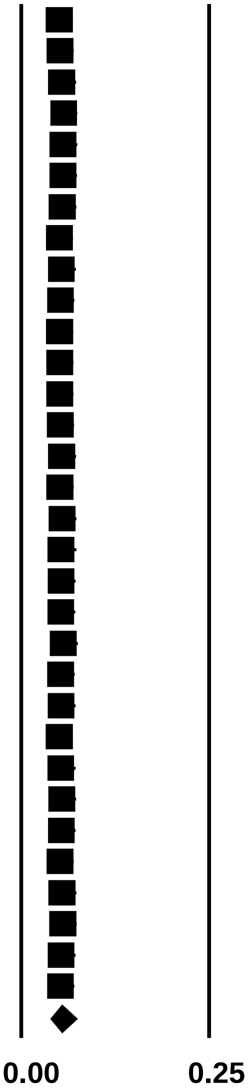


Figure S3. Leave-one-out sensitivity analysis of the pooled seizure occurrence proportion after solid organ transplantation. Pooled estimates and 95% confidence intervals were recalculated after sequential omission of each independent cohort. The included studies are cited in References [12,16,26–55].

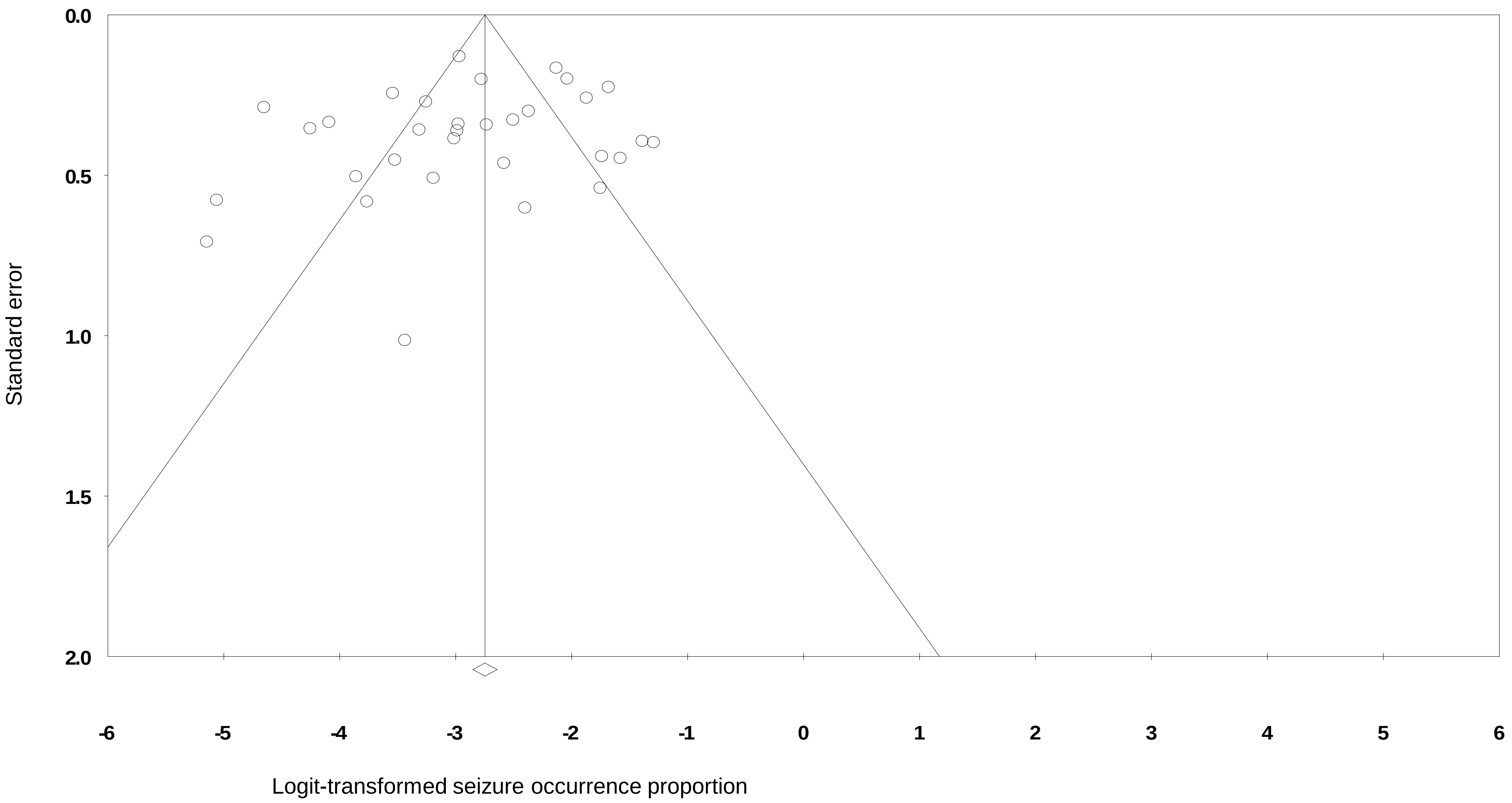


Figure S4. Funnel plot of the 32 independent cohorts included in the primary meta-analysis of seizure occurrence after solid organ transplantation. The horizontal axis represents the logit-transformed seizure occurrence proportion, and the vertical axis represents its standard error.

Supplementary Table S1: Detailed Joanna Briggs Institute risk-of-bias assessment of the included reports

| Study | Organ | Population | Study design | Total N | Seizure n | Q1 Sample frame | Q2 Sampling | Q3 Sample size | Q4 Subjects/setting | Q5 Coverage | Q6 Valid seizure identification | Q7 Standard measurement | Q8 Statistical analysis | Q9 Follow-up/completeness | Yes count | Overall risk of bias |
|---|---|---|---|---|---|---|---|---|---|---|---|---|---|---|---|---|
| **Ameres et al. [32]** | Liver | Pediatric infants <24 months | Retrospective cohort | 40 | 8 | Yes | Yes | Yes | Yes | Yes | Yes | Unclear | Yes | Yes | 8 | Low |
| **Appiani et al. [53]** | Liver | Adult LT recipients | Retrospective observational study / risk model | 376 | 40 | Yes | Yes | Yes | Yes | Yes | Yes | Yes | Yes | Yes | 9 | Low |
| **Avorio et al. [52]** | Liver | Orthotopic liver transplantation recipients | Single-center retrospective study | 376 | 14 | Yes | Yes | Yes | Yes | Yes | Yes | Yes | Yes | Unclear | 8 | Low |
| **Berry et al. [40]** | Liver | Adult | Retrospective cohort using institutional registry | 1265 | 12 | Yes | Yes | Yes | Yes | Unclear | Unclear | Unclear | Yes | Unclear | 5 | Moderate |
| **Cengiz et al. [49]** | Kidney | Renal transplantation patients | Retrospective clinical study | 344 | 2 | Yes | Yes | Yes | Unclear | Unclear | Unclear | Unclear | Yes | Yes | 5 | Moderate |
| **Christian et al. [55]** | Liver | Adult LDLT recipients | Single-center cohort / predictive model | 569 | 8 | Yes | Yes | Yes | Yes | Yes | Yes | Unclear | Yes | Yes | 8 | Low |
| **Colla Machado et al. [26]** | Heart | Adult | Retrospective single-center cohort | 193 | 4 | Yes | Yes | Yes | Yes | Yes | Unclear | Unclear | Yes | Yes | 7 | Low |
| **Derle et al. [12]** | Liver | Adult | Retrospective cohort / seizure-focused | 146 | 23 | Yes | Yes | Yes | Unclear | Unclear | Unclear | Yes | Yes | Unclear | 5 | Moderate |
| **Dhar et al. [41]** | Liver | Adult | Retrospective cohort | 101 | 4 | Yes | Yes | Yes | Yes | Yes | Unclear | Unclear | Yes | Yes | 7 | Low |
| **Drake et al. [16]** | Kidney | Pediatric kidney transplant recipients | Retrospective case-control within single-center cohort | 36 | 3 | Yes | Yes | No | Yes | Yes | Yes | Yes | Yes | Unclear | 7 | Low |
| **Elwan et al. [39]** | Liver | Adult | Prospective observational cohort | 35 | 6 | Yes | Yes | No | Yes | Yes | Yes | Unclear | Yes | Yes | 7 | Low |
| **Erol et al. [37]** | Liver | Pediatric | Retrospective single-center cohort | 40 | 6 | Yes | Yes | No | Yes | Yes | Yes | Yes | Yes | Yes | 8 | Low |
| **Erol et al. [56]** | Liver | Pediatric Wilson disease | Retrospective case series/cohort | 17 | 6 | Yes | Yes | No | Yes | Yes | Yes | Yes | Yes | Yes | 8 | Low |

| | | | | | | | | | | | | | | | | |
|---|---|---|---|---|---|---|---|---|---|---|---|---|---|---|---|---|
| **Fernandez et al. [45]** | Liver and combined liver-bowel | Pediatric liver / combined liver and small bowel transplant episodes | Single tertiary-center audit/retrospective study | 127 | 17 | Yes | Yes | Yes | Yes | Unclear | Unclear | Unclear | Yes | Yes | 6 | Moderate |
| **Fernández-Ramos et al. [47]** | Solid organ: pediatric cardiac, hepatic, pulmonary | Pediatric solid organ transplant recipients | Retrospective descriptive study | 140 | 12 | Unclear | Yes | Yes | Yes | Unclear | Unclear | Unclear | Yes | Yes | 5 | Moderate |
| **Fu et al. [42]** | Liver | Adult | Retrospective review | 227 | 8 | Yes | Yes | Yes | Yes | Yes | Unclear | Unclear | Yes | Yes | 7 | Low |
| **Gungor et al. [30]** | Liver | Pediatric | Retrospective cohort | 242 | 28 | Yes | Yes | Yes | Yes | Yes | Unclear | Unclear | Yes | Unclear | 6 | Moderate |
| **Kanamori et al. [34]** | Liver | Pediatric | Retrospective observational cohort | 602 | 17 | Yes | Yes | Yes | Yes | Unclear | Unclear | Unclear | Yes | Unclear | 5 | Moderate |
| **Kang et al. [51]** | Liver | LT recipients; nested case-control for seizures | Retrospective cohort with nested case-control | 1243 | 61 | Yes | Yes | Yes | Yes | Yes | Yes | Yes | Yes | Yes | 9 | Low |
| **Khalil et al. [36]** | Liver | Adult | Prospective/observational ICU cohort | 149 | 7 | Yes | Yes | Yes | Yes | Yes | Yes | Unclear | Yes | Yes | 8 | Low |
| **Kim et al. [54]** | Lung | Lung transplant recipients | Retrospective cohort; PRES-focused | 147 | 9 | Unclear | Yes | Yes | Yes | Yes | Yes | Unclear | Yes | Yes | 7 | Low |
| **Li et al. [29]** | Liver | Mixed/unclear | Retrospective cohort | 474 | 3 | Yes | Yes | Yes | Unclear | Unclear | Unclear | Unclear | Yes | Unclear | 4 | High |
| **Menon et al. [35]** | Liver | Pediatric | Retrospective cohort | 71 | 5 | Yes | Yes | Yes | Yes | Yes | Unclear | Unclear | Yes | Yes | 7 | Low |
| **Navarro et al. [27]** | Heart | Adult | Retrospective cohort | 166 | 8 | Yes | Yes | Yes | Yes | Yes | Yes | Unclear | Yes | Yes | 8 | Low |
| **Orgun et al. [28]** | Heart | Pediatric | Retrospective single-center cohort | 37 | 8 | Yes | Yes | No | Yes | Yes | Unclear | Unclear | Yes | Unclear | 5 | Moderate |
| **Piñero et al. [31]** | Liver | Adult + pediatric | Single-center cohort | 443 | 26 | Yes | Yes | Yes | Yes | Yes | Unclear | Unclear | Yes | Yes | 7 | Low |
| **Saner et al. [33]** | Liver | Adult/mixed LT ICU recipients | Retrospective cohort | 174 | 5 | Yes | Yes | Yes | Yes | Yes | Unclear | Unclear | Yes | Yes | 7 | Low |
| **Saner et al. [57]** | Liver | Adult LT recipients admitted to ICU | Retrospective cohort; severe neurologic events | 168 | 9 | Yes | Yes | Yes | Yes | Yes | Unclear | Unclear | Yes | Yes | 7 | Low |
| **Varghese et al. [43]** | Liver | LT recipients on tacrolimus; | Retrospective adverse- | 32 | 1 | Unclear | Yes | No | Yes | Yes | Yes | Unclear | Yes | Yes | 6 | Moderate |

| | | | | | | | | | | | | | | | | |
|---|---|---|---|---|---|---|---|---|---|---|---|---|---|---|---|---|
| | | selected low-comorbidity cohort | effect/TDM study | | | | | | | | | | | | | |
| **Xie et al. [44]** | Liver | Pediatric LT recipients | Single-center retrospective cohort | 27 | 4 | Yes | Yes | No | Yes | Yes | Yes | Yes | Yes | Yes | 8 | Low |
| **Yardimci et al. [48]** | Kidney | Adult renal transplant recipients | Retrospective cohort | 132 | 3 | Yes | Yes | Yes | Yes | Yes | Yes | Unclear | Yes | Yes | 8 | Low |
| **Yavuz et al. [50]** | Lung | Lung transplant recipients with neurologic symptoms typical of PRES | Retrospective cohort + nested comparison | 545 | 9 | Unclear | Yes | Yes | Yes | Unclear | Yes | Unclear | Yes | Unclear | 5 | Moderate |
| **Yuksel et al. [38]** | Liver | Adult/mixed | Retrospective cohort | 185 | 9 | Yes | Yes | Yes | Yes | Unclear | Unclear | Unclear | Yes | Yes | 6 | Moderate |
| **Zivkovic et al. [46]** | Lung | Adult lung allograft recipients | Retrospective cohort | 132 | 10 | Yes | Yes | Yes | Yes | Yes | Unclear | Unclear | Yes | Yes | 7 | Low |

## Supplementary Table S2. Complete search strategies for PubMed, Embase, and Web of Science

### 1) PubMed

| | |
|---|---|
| **Search date** | 28 February 2026 |
| **Date limits** | Publication date: 1 January 2000–31 December 2025 |
| **Language** | No language restriction |
| **Search strategy** | (("Seizures"[Mesh] OR seizure*[tiab] OR epilep*[tiab] OR convulsion*[tiab] OR "status epilepticus"[tiab])OR("neurologic complication*"[tiab] OR "neurological complication*"[tiab] OR neurotoxicity[tiab] OR "posterior reversible encephalopathy syndrome"[tiab] OR PRES[tiab]))AND("Organ Transplantation"[Mesh] OR "Liver Transplantation"[Mesh] OR "Kidney Transplantation"[Mesh]OR "Heart Transplantation"[Mesh] OR "Lung Transplantation"[Mesh]OR "solid organ transplant*"[tiab] OR "liver transplant*"[tiab] OR "kidney transplant*"[tiab]OR "renal transplant*"[tiab] OR "heart transplant*"[tiab] OR "lung transplant*"[tiab])AND ("2000/01/01"[Date - Publication] : "2025/12/31"[Date - Publication]) |

### 2) Embase

| | |
|---|---|
| **Search date** | 28 February 2026 |
| **Date limits** | Publication date: 1 January 2000–31 December 2025 |
| **Language** | No language restriction |
| **Search strategy** | ('seizure'/exp OR seizure*:ti,ab,kw OR 'epilepsy'/exp OR epilep*:ti,ab,kw OR convulsion*:ti,ab,kw OR 'status epilepticus'/exp OR 'status epilepticus':ti,ab,kw OR 'neurologic complication*':ti,ab,kw OR 'neurological complication*':ti,ab,kw OR neurotoxicity:ti,ab,kw OR 'posterior reversible encephalopathy syndrome'/exp OR 'posterior reversible encephalopathy syndrome':ti,ab,kw OR pres:ti,ab,kw ) AND ('organ transplantation'/exp OR 'liver transplantation'/exp OR 'kidney transplantation'/exp OR 'heart transplantation'/exp OR 'lung transplantation'/exp OR 'solid organ transplant*':ti,ab,kw OR 'liver transplant*':ti,ab,kw OR 'kidney transplant*':ti,ab,kw OR 'renal transplant*':ti,ab,kw OR 'heart transplant*':ti,ab,kw OR 'lung transplant*':ti,ab,kw) |

### 3) Web of Science

| | |
|---|---|
| **Search date** | 28 February 2026 |
| **Date limits** | Timespan: 2000–2025 |
| **Language** | No language restriction |
| **Search strategy** | TS=((seizure* OR epilep* OR convulsion* OR "status epilepticus" OR "neurologic complication*" OR "neurological complication*" OR neurotoxicity OR "posterior reversible encephalopathy syndrome" OR PRES) AND ("solid organ transplant*" OR "liver transplant*" OR "kidney transplant*" OR "renal transplant*" OR "heart transplant*" OR "lung transplant*")) |

## Supplementary Table S3. Reports that appeared potentially eligible but were excluded after full-text assessment

*This table lists selected reports that could reasonably appear to meet the eligibility criteria and is not an exhaustive list of all reports excluded after full-text assessment.*

| Full citation | Reason for exclusion |
|---|---|
| 76. Acharya C, White MB, Fagan A, et al. Liver Transplant Is Associated with Sustained Improvement in Tandem Gait and Risk of Falls. Dig Dis Sci. 2021;66(4):1360–1366. doi:10.1007/s10620-020-06261-y. | Post-transplant neurological/cognitive and gait outcomes were evaluated, but seizure-specific occurrence data were not reported. |
| 77. Al-Sarraf AJ, Haque M, Pudek M, Yoshida EM. Central pontine myelinolysis after orthotopic liver transplant—a rare complication. Exp Clin Transplant. 2010;8(4):321–324. | Single-patient case report; no defined transplant cohort denominator for estimating seizure occurrence. |
| 78. Alba AC, Verocai Flaman F, Granton J, Delgado DH. Patent foramen ovale does not have a negative impact on early outcomes in patients undergoing liver transplantation. Clin Transplant. 2011;25(1):151–155. doi:10.1111/j.1399-0012.2010.01210.x. | Neurological complications were reported only as an aggregate outcome; seizure-specific data were not available. |
| 79. Alexopoulos SP, Matsuoka L, Hafberg E, Morgan T, Thurm C, Hall M, Godown J. Liver Transplantation for Propionic Acidemia: A Multicenter-linked Database Analysis. J Pediatr Gastroenterol Nutr. 2020;70(2):178–182. doi:10.1097/MPG.0000000000002534. | Neurological outcomes were clinically relevant to the underlying disease, but post-transplant seizure occurrence was not reported separately. |
| 80. Alonso EM, Martz K, Wang D, et al. Factors Predicting Health Related Quality of Life in Pediatric Liver Transplant Recipients in the Functional Outcomes Group. Pediatr Transplant. 2013;17(7):605–611. doi:10.1111/petr.12126. | Anti-seizure medication use was analyzed as a predictor of health-related quality-of-life/cognitive outcomes, but post-transplant seizure occurrence was not reported as an outcome. |
| 81. Barreiros AP, Post F, Hoppe-Lotichius M, et al. Liver transplantation and combined liver-heart transplantation in patients with familial amyloid polyneuropathy: a single-center experience. Liver Transpl. 2010;16(3):314–323. doi:10.1002/lt.21996. | Seizures were described as pre-transplant manifestations in selected patients; post-transplant seizure occurrence was not reported for the transplant cohort. |
| 82. Bauer J, Thul J, Krämer U, et al. Heart transplantation in children and infants: short-term outcome and long-term follow-up. Pediatr Transplant. 2001;5(6):457–462. doi:10.1034/j.1399-3046.2001.00023.x. | Post-transplant neurological morbidity was reported as a composite neurological-deficit outcome without separate seizure-specific data. |
| 83. Belliere J, Rolland M, Tournier E, et al. Early necrotic skin lesions after a ABO-incompatible kidney transplantation: The threat of Cunninghamella Spp. Transpl Infect Dis. 2019;21(6):e13173. doi:10.1111/tid.13173. | Single-patient case report describing post-transplant generalized tonic-clonic seizures; no defined transplant cohort denominator. |
| 84. Clissold R, Bingham C. Late presentation of toxoplasmosis in renal transplant recipients. NDT Plus. 2010;3(5):480–482. doi:10.1093/ndtplus/sfq113. | Two-patient case report/series; no defined transplant cohort denominator for estimating post-transplant seizure occurrence. |
| 85. De Giorgio R, Pironi L, Rinaldi R, et al. Liver transplantation for mitochondrial neurogastrointestinal encephalomyopathy. Ann Neurol. 2016;80(3):448–455. doi:10.1002/ana.24724. | Single-patient case report; no cohort-level post-transplant seizure occurrence data. |

Note: Exclusion reasons were refined from the full-text screening record to identify the specific eligibility criterion that was not met. Aggregate full-text exclusion counts are reported separately in the PRISMA flow diagram and Results.

## Supplementary Table S4. Additional subgroup, meta-regression, robustness, and follow-up ascertainment analyses

### Panel A. Formal subgroup comparisons

| Comparison | Groups (k) | Pooled seizure occurrence, % (95% CI) | Qbetween | df | p value |
|---|---|---|---|---|---|
| Transplant organ | Liver (21); Heart (3); Kidney (3); Lung (3) | Liver 5.4 (3.7–7.7); Heart 6.4 (1.6–22.0); Kidney 2.3 (0.5–9.6); Lung 4.3 (1.7–10.6) | 1.443 | 3 | 0.695 |
| Recipient age | Adult (13); Pediatric (10) | Adult 4.4 (2.5–7.5); Pediatric 10.7 (7.0–16.2) | 6.490 | 1 | 0.011 |
| Recipient age within liver-only cohorts | Adult liver (9); Pediatric liver (6) | Adult liver 4.6 (2.2–9.4); Pediatric liver 9.9 (5.1–18.6) | 2.342 | 1 | 0.126 |
| Follow-up / seizure ascertainment window | Early/index-hospitalization (11); Extended/long-term (11); Unclear/variable (10) | Early/index-hospitalization 4.7 (2.7–8.3); Extended/long-term 6.2 (4.2–9.1); Unclear/variable 5.0 (2.5–9.7) | 0.695 | 2 | 0.707 |

### Panel B1. Exploratory univariable meta-regression: model summary

| Moderator | k | Effect / omnibus test | p value | $R^2$ analog | Residual $I^2$ (%) | Reference category (categorical moderators) |
|---|---|---|---|---|---|---|
| Publication year | 32 | β = −0.0254 (95% CI, −0.0779 to 0.0270) | 0.3415 | 0.00 | 86.77 | — |
| Cohort size, ln(N) | 32 | β = −0.6218 (95% CI, −0.8806 to −0.3630) | <0.001 | 0.31 | 81.58 | — |
| Cohort size, raw N (sensitivity) | 32 | β = −0.0016 (95% CI, −0.0025 to −0.0007) | 0.0004 | 0.15 | 83.86 | — |
| Follow-up / ascertainment category | 32 | Q = 0.44 (df = 2) | 0.8010 | 0.00 | 87.75 | Early/index-hospitalization |
| Geographic region | 32 | Q = 4.07 (df = 3) | 0.2535 | 0.01 | 85.64 | Americas |
| Recipient age-group classification | 32 | Q = 12.78 (df = 2) | 0.0017 | 0.07 | 85.25 | Pediatric |
| Transplant type | 32 | Q = 3.93 (df = 4) | 0.4155 | 0.00 | 87.62 | Heart |
| Study design | 32 | Q = 1.67 (df = 2) | 0.4345 | 0.00 | 87.55 | Retrospective |

### Panel B2. Category-specific coefficients for categorical moderators

| Moderator | Comparison with reference | β | 95% CI | Z | p value |
|---|---|---|---|---|---|
| Follow-up / ascertainment | Extended/long-term vs early/index-hospitalization | 0.2622 | −0.5252 to 1.0496 | 0.65 | 0.5139 |
| | Unclear/variable vs early/index-hospitalization | 0.0846 | −0.7211 to 0.8903 | 0.21 | 0.8369 |
| Geographic region | East/South Asia vs Americas | −0.1754 | −1.0549 to 0.7041 | −0.39 | 0.6959 |
| | Europe vs Americas | 0.3196 | −0.5645 to 1.2036 | 0.71 | 0.4786 |
| | West Asia/North Africa vs Americas | 0.6422 | −0.2036 to 1.4879 | 1.49 | 0.1367 |
| Recipient age group | Adult vs Pediatric | −0.9643 | −1.6740 to −0.2546 | −2.66 | 0.0077 |
| | Mixed/unspecified vs Pediatric | −1.3665 | −2.1515 to −0.5815 | −3.41 | 0.0006 |

| Moderator | Comparison with reference | β | 95% CI | Z | p value |
|---|---|---|---|---|---|
| Transplant type | Kidney vs Heart | −1.0389 | −2.5917 to 0.5139 | −1.31 | 0.1898 |
| | Liver vs Heart | −0.1983 | −1.2993 to 0.9027 | −0.35 | 0.7241 |
| | Lung vs Heart | −0.4348 | −1.8654 to 0.9958 | −0.60 | 0.5514 |
| | Mixed vs Heart | 0.5577 | −1.0147 to 2.1301 | 0.70 | 0.4870 |
| Study design | Other/unclear vs Retrospective | −0.5932 | −1.8493 to 0.6629 | −0.93 | 0.3547 |
| | Prospective vs Retrospective | 0.5637 | −0.7618 to 1.8893 | 0.83 | 0.4045 |

## Panel C. Robustness and selected-population sensitivity analyses

| Analysis | k | Pooled seizure occurrence, % | 95% CI (%) | $\tau^2$ | $I^2$ (%) | 95% PI (%) |
|---|---|---|---|---|---|---|
| REML with Hartung–Knapp adjustment | 32 | 5.3 | 3.7–7.4 | 0.828 | — | — |
| Excluding all seizure- or PRES-focused cohorts | 29 | 5.3 | 3.9–7.1 | 0.597 | 86.1 | 1.1–21.9 |

## Panel D. Follow-up/seizure ascertainment classification of the 32 independent cohorts

| Category | Cohorts | k |
|---|---|---|
| Early/index-hospitalization | Appiani; Avorio; Berry; Colla Machado; Dhar; Drake; Erol; Fu; Khalil; Saner; Xie | 11 |
| Extended/long-term | Christian; Elwan; Fernandez; Gungor; Kang; Menon; Piñero; Varghese; Yardimci; Yuksel; Zivkovic | 11 |
| Unclear/variable | Ameres; Cengiz; Derle; Fernández-Ramos; Kanamori; Kim; Li; Navarro; Orgun; Yavuz | 10 |

Notes: Follow-up classification was based on the seizure ascertainment window used for the quantitative outcome rather than on overall clinical follow-up. Early/index-hospitalization indicated seizure ascertainment restricted to ≤30 days after transplantation or to the index transplant hospitalization without explicit later post-discharge ascertainment; extended/long-term indicated explicit seizure ascertainment beyond 30 days that included later post-transplant follow-up; and unclear/variable indicated that the seizure ascertainment window could not be reliably determined. Studies restricted to the index hospitalization remained in the early/index-hospitalization category regardless of actual hospitalization duration unless later post-discharge seizure ascertainment was explicitly reported. For Xie et al., seizure ascertainment was restricted to the first 2 weeks, whereas the reported mean follow-up of 33.7 months referred to overall cohort follow-up. All exploratory univariable meta-regression models included 32 independent cohorts and were fitted on the logit event-rate scale using random-effects meta-regression with the method-of-moments (MM) estimator for $\tau^2$. Regression coefficients and 95% CIs were calculated using Z-distribution-based inference, with two-sided Z tests for individual coefficients and omnibus Q tests for categorical moderators. Meta-regression analyses were exploratory and represent study-level rather than patient-level associations. Formal subgroup comparisons used mixed-effects models with separate estimates of between-study variance across subgroups. Abbreviations: CI, confidence interval; df, degrees of freedom; MM, method of moments; PI, prediction interval; PRES, posterior reversible encephalopathy syndrome; REML, restricted maximum likelihood.
